# Melatonin alleviates pyroptosis by regulating the SIRT3/FOXO3α/ROS axis and interacting with apoptosis in Atherosclerosis progression

**DOI:** 10.1186/s40659-023-00479-6

**Published:** 2023-12-02

**Authors:** Lin Cong, Xiankun Liu, Yiming Bai, Qin Qin, Lili Zhao, Ying Shi, Yunpeng Bai, Zhigang Guo

**Affiliations:** 1 Academy of Medical Engineering and Translational Medicine, Tianjin University, Tianjin, China; 2 Tianjin Institute of Cardiovascular Diseases, Chest Hospital, Tianjin University, Tianjin, China; 3 Department of Cardiac Surgery, Chest Hospital, Tianjin University, Tianjin, China; 4 Clinical School of Thoracic, Tianjin Medical University, Tianjin, China; 5 Tianjin Key Laboratory of Cardiovascular Emergency and Critical Care, Tianjin Municipal Science and Technology Bureau, Tianjin, China

**Keywords:** Pyroptosis, Melatonin (MT), SIRT3/FOXO3α/ROS axis, Apoptosis, Atherosclerosis (AS)

## Abstract

**Background:**

Atherosclerosis (AS), a significant contributor to cardiovascular disease (CVD), is steadily rising with the aging of the global population. Pyroptosis and apoptosis, both caspase-mediated cell death mechanisms, play an essential role in the occurrence and progression of AS. The human pineal gland primarily produces melatonin (MT), an indoleamine hormone with powerful anti-oxidative, anti-pyroptotic, and anti-apoptotic properties. This study examined MT’s anti-oxidative stress and anti-pyroptotic effects on human THP-1 macrophages treated with nicotine.

**Methods:**

In vitro, THP-1 macrophages were induced by 1 µM nicotine to form a pyroptosis model and performed 30 mM MT for treatment. In vivo, ApoE^-/-^ mice were administered 0.1 mg/mL nicotine solution as drinking water, and 1 mg/mL MT solution was intragastric administrated at 10 mg/kg/day. The changes in pyroptosis, apoptosis, and oxidative stress were detected.

**Results:**

MT downregulated pyroptosis, whose changes were paralleled by a reduction in reactive oxygen species (ROS) production, reversal of sirtuin3 (SIRT3), and Forkhead box O3 (FOXO3α) upregulation. MT also inhibited apoptosis, mainly caused by the interaction of caspase-1 and caspase-3 proteins. Vivo studies confirmed that nicotine could accelerate plaque formation. Moreover, mice treated with MT showed a reduction in AS lesion area.

**Conclusions:**

MT alleviates pyroptosis by regulating the SIRT3/FOXO3α/ROS axis and interacting with apoptosis. Importantly, our understanding of the inhibitory pathways for macrophage pyroptosis will allow us to identify other novel therapeutic targets that will help treat, prevent, and reduce AS-associated mortality.

**Supplementary Information:**

The online version contains supplementary material available at 10.1186/s40659-023-00479-6.

## Introduction

Atherosclerosis (AS), which is a chronic inflammatory disease of the arterial wall characterized by plaque rupture, stenosis, or occlusion due to platelet aggregation and thrombosis, is the leading cause of cardiovascular disease (CVD) [1, 2]. According to the China Cardiovascular Health and Disease Report 2021, the morbidity and mortality of cardiovascular disease still rank first in China, and it is estimated that there are 330 million people with this disease [3]. As a critical component of inflammatory plaques, macrophages play a vital role in developing atherosclerotic cardiovascular disease (ASCVD) [4, 5]. Pyroptosis, a highly inflammatory form of programmed cell death (PCD) typically triggered in the vascular wall, increases plaque instability and accelerates AS progression [6]. The expression of proinflammatory factors in humans has been associated with AS lesions, especially infiltrating macrophages [7]. Notably, this complex and diverse mechanism warrants additional research and investigation. Consequently, therapies that inhibit macrophage pyroptosis and inflammation may reduce AS-related morbidity and mortality.

PCD pathways are required for cell turnover and tissue homeostasis, and their dysregulation is implicated in aging and age-related diseases [8]. A growing body of evidence suggests that different PCD pathways are interrelated at multiple levels and may affect CVD. Pyroptosis is a form of proinflammatory PCD mediated by a multiprotein complex called the inflammasome [9]. In the classic pyroptosis pathway, NOD-like receptor family protein 3 (NLRP3) recruits the adapter protein ASC (apoptosis-associated speck-like protein containing a C-terminal caspase recruitment domain), which results in the cleavage of pro-caspase-1 into biologically active caspase-1 and the maturation of interleukin-1β (IL-1β) and interleukin-18 (IL-18) [10, 11]. During AS, various damaging cardiovascular factors can trigger inflammation and exacerbate atheroma formation by activating the NLRP3 inflammasome, and inhibiting the inflammasome contributes to plaque stabilization [12]. Apoptosis and pyroptosis are PCD processes characterized by chromatin condensation, nuclear condensation, and caspase dependence. Consistent with pyroptosis, apoptosis is an independent risk factor for AS lesions and is vital in promoting unstable plaque formation [13]. Few studies have investigated the relationship between apoptosis and pyroptosis; however, several essential proteins are known to bridge the two forms of cell death, such as the interactions between GSDMD and caspase-1 [14], GSDME and caspase-3 [15], and FADD and caspase-8 [16–19].

Mitochondrial ROS have been suggested to be a critical activator of the NLRP3 inflammasome complex, and ROS inhibition can also be exploited to prevent pyroptosis in AS and other diseases [20]. SIRT3 and Forkhead box O3 (FOXO3α) are human longevity-associated genes [21, 22]. Sirtuin3 (SIRT3) is a member of a family of proteins with NAD^+^-dependent protein deacetylase activity that regulates many critical cellular processes, including transcription, metabolism, and oxidative stress responses [23, 24]. Our previous study showed that SIRT3, a critical mitochondrial deacetylase, could promote mitophagy, inhibit ROS production, and prevent NLRP3 inflammasome activation to protect macrophages from stress [25]. In addition, the transcription factor FOXO3α has been shown to play a complex role in oxidative stress. This factor can not only induce apoptosis in cells under oxidative stress but also inhibit the occurrence of oxidative stress [26, 27]. The pathways through which FOXO3 regulates oxidative stress in cells are very complex. The known pathways include the PI3K-AKT-FoxO3α pathway, SIRT-FOXO3α pathway, and AMPK-FOXO3α-MnSOD pathway [28]. This study examined whether FOXO3α and SIRT3 were independent or synergistic in preventing NLRP3 activation by regulating oxidative stress.

Melatonin (N-acetyl-5-methoxytryptamine, MT) is a neuroendocrine hormone synthesized in the pineal gland and many other organs. It has been reported that MT has an antioxidant effect, and many in vitro and in vivo studies, as well as some clinical experiments, have been conducted to determine whether the antioxidant activity of MT has a beneficial effect on CVD by modulating oxidative stress. Previous research has demonstrated that MT positively inhibits both pyroptosis and apoptosis [9, 29]. However, no study has described the interaction between the two modes of cell death inhibited by MT and the specific molecular mechanisms involved in this inhibition. In this study, we focused on the specific mechanism by which MT inhibited nicotine-induced pyroptosis in THP-1 macrophages and investigated whether there was a mutual regulatory mechanism between pyroptosis and apoptosis.

## Materials and methods

### Cell culture and experimental treatments

THP-1 cells were purchased from Procell (Wuhan, China). THP-1 monocytes (1.0 × 10^5^ cells/mL) were added dropwise into six-well plates with complete medium containing 100 ng/ml phorbol-12-myristate-13-acetate (PMA; Sigma Chemical Co. St. Louis, MO, USA) and incubated for 48 h. THP-1 macrophages were treated with 1 µM nicotine for 24 h. In addition, drugs and inhibitors, such as n-acetyl cysteine (NAC, 1 mM), 3-TYP (50 µM), VX-765 (10 µM) and Z-DEVD-FMK (100 µM), were administered before MT (30 mM).

### Establishment of the in vivo AS model

This study was approved by the Laboratory Animal Ethics Committee of Tianjin Chest Hospital on January 5, 2023 (registration number: TJCH-2003-001). All animal experiments were performed according to procedures approved by the Laboratory Animal Ethics Committee of Tianjin Chest Hospital and complied with the ARRIVE (Animal Research: Reporting of In Vivo Experiments) guidelines and regulations.

Ten-week-old male ApoE^−/−^ mice (weighing 25.5–29.5 g) were divided into six groups of 10 mice each: the standard diet group (NCD), the standard diet group treated with nicotine (NCD + nicotine), the standard diet group treated with nicotine and MT (NCD + nicotine + MT), the high-fat diet (0.15% cholesterol and 21% fat) (Huafukang, Beijing, China) group (HFD), the high-fat diet group treated with nicotine and MT (HFD + nicotine + MT), and the high-fat diet group treated with nicotine (HFD + nicotine). Each mouse was given an average of 8 g of feed and 12 mL of water daily. The mice in the HFD group were maintained on a high-fat diet for 12 weeks to establish the AS mouse model. The NCD + nicotine and HFD + nicotine groups were also treated for 12 weeks to observe the formation of atherosclerotic plaques. Nicotine was dissolved in distilled water at a concentration of 0.1 mg/mL and was given in drinking water for 12 weeks. Dissolve MT in distilled water to a 1 mg/mL concentration, and the solvent will not affect subsequent experiments. According to the literature [9], 10 mg/kg/day daily was intragastric administration for 12 weeks.

### Cell viability assay

THP-1 macrophages were exposed to nicotine/MT. Then, 100 µL of medium containing 10% CCK-8 (Yeason, Shanghai, China) was added to each well. After incubation for 1 h in the dark, the absorption at a wavelength of 450 nm was measured using a microplate reader (Varian Australia Pty Ltd, Australia).

### Enzyme-linked immunosorbent assay (ELISA)

The supernatant of THP-1 macrophages was collected to detect soluble IL-18, IL-1β, and 4-HNE; THP-1 macrophage lysate was collected to detect soluble Caspase-1 and Caspase-3; and mouse serum was collected to detect IL-18, IL-1β, and testosterone levels by using ELISA kits (Elabscience Biotechnology Co. Ltd., Wuhan, China).

### Immunofluorescence analysis

THP-1 macrophages were fixed with 4% paraformaldehyde for 30 min and permeabilized with 1% Triton X-100 for 20 min. The macrophages were blocked with 3% BSA and incubated with NLRP3, ASC and IL-18 antibodies overnight at 4 °C. Finally, the macrophages were incubated with fluorescence-labelled secondary antibodies at 37 ℃ for 1 h in the dark. DAPI staining was used to counterstain the nucleus. Fluorescence intensities were quantified with a laser scanning confocal microscope (Nikon, Japan).

### Reactive oxygen species (ROS) analysis

Treated cells were incubated with the fluorescent dye 2′-7′-dichlorofluorescein diacetate (DCFH-DA)/MitoSOX Red Mitochondrial Superoxide Indicator for 30 min at 37 °C in the dark. The cells were analyzed by fluorescence spectrophotometry (Nikon, Japan) or flow cytometry (Agilent, America).

### Western blot (WB) analysis

The membranes were blocked with 5% nonskimmed dry milk containing 0.05% Tween 20 in Tris-buffered saline for 1.5 h. The membrane was probed with primary antibodies against NLRP3 (proteintech), ASC (Omnimabs), pro-caspase-1/caspase-1 (Abcam), IL-18 (proteintech) and IL-1β (proteintech), GAPDH (proteintech), FOXO3α (proteintech), SIRT3 (proteintech), mnSOD (proteintech), 4-HNE (Abcam), Catalase (proteintech), Caspase-3 (proteintech), GSDMD (proteintech), GSDME (proteintech), all diluted at 1:1000 in 5%BAS-TBST buffer 4 °C overnight. This was followed by incubation with horseradish peroxidase (HRP)-conjugated secondary antibody (1:5000; proteintech) for 1.5 h. The immunoblots were visualized by chemiluminescence using an imaging system (Bio-Rad, America). Protein bands were quantified using Quantity One software (Bio-Rad Laboratories, Hercules, CA, USA). Due to the need to display the results of proteins with different molecular weights on the same PVDF membrane, we performed imprinting cleavage before antibody hybridization. To ensure the rigor of the experiment, we cut the blots of the exact result graph of the same grouping on a PVDF membrane. However, the exposure time of different bands is inconsistent due to differences in antibodies. Therefore, we adopted a separate color development method (simultaneous color development may lead to underexposure/overexposure of some blots).

### Quantitative PCR (q-RT‒PCR)

The UNIQ-10 Columnar Total RNA Extraction Kit (Sangon Biotech, Shanghai, China) was used for RNA extraction, and reverse transcription was performed using the RT Easy II First Strand cDNA Synthesis Kit (FORGENE, Sichuan, China). Then, 1 µL of cDNA was amplified using Real-Time PCR Easy (SYBR Green I) (FORGENE, Sichuan, China) on an ABI 7900HT Sequence Detection System (ABI Applied Biosystems, Foster City, CA, USA). Table 1 shows the primer sequences.

**Table 1:** Primer sequences

Species	Gene	Forward Primer	Reverse Primer
Human	NLRP3	CACCTGTTGTGCAATCTGAAG	GCAAGATCCTGACAACATGC
Human	ASC	AGGCCTGCACTTTATAGACC	GCTGGTGTGAAACTGAAGAG
Human	Caspase-1	CCTTAATATGCAAGACTCTCAAGGA	TAAGCTGGGTTGTCCTGCACT
Human	IL-18	TGCATCAACTTTGTGGCAAT	ATAGAGGCCGATTTCCTTGG
Human	IL-1β	TACCTGTCCTGCGTGTTGAA	TCTTTGGGTAATTTTTGGGATCT
Human	SIRT3	GAGCCTTTTGCCAGCTTGTCT	CATCCCCTAGCTGGACCACAT
Human	mnSOD	CAATAGAAGGCTGCCCTTTC	CACAGTGCACAGGAACACAG
Human	FOXO3α	GCAAAGCAGACCCTCAAACTG	GCGTGGGATTCACAAAGGTG

### Transfection of small interfering RNAs (siRNAs)

Five microlitres of siRNA (20 µM) and 8 µL of Hieff TransTM siRNA (Yeason, Shanghai, China) were mixed with 200 µL of RPMI 1640 medium. The mixture was added to individual THP-1 macrophage cultures in a six-well plate containing fresh medium and incubated for 48 h. The caspase-1 siRNA duplexes S1 (sense: CCUGUGAUGUGGAGGAAAUTT; and anti sense: AUUUCCUCCACAUCAXAGGTT), S2 (sense: UGG AAGACUCAUUGAACAUTT, anti sense: AUGUUCA AUGAGUCUUCCATT), S3 (sense: GAAGACUCAU UGAACAUAUTT, anti sense: AUAUGUUCAAU GAGUCUUCTT) were used. The FOXO3α siRNA duplexes S1 (sense: GCUGUCUCCAUGGACAAUATT, anti-sense: UAUUGUCCAUGGAGACAGCTT), S2 (sense: GCUCACUUCGGACUCACUUTT, anti-sense: AAGUGAGUCCGAAGUGAGCTT), and S3 (sense: CCUCAUCUCCACACAGAAUTT; and anti-sense: AUUCUGUGUGGAGAUGAGGTT) were used.

### Lentiviral transduction

THP-1 macrophages were inoculated, and the virus was added according to the previously determined multiplicity of infection (MOI). The cells were randomly divided into blank control and overexpression groups. The blank control group was cultured with ordinary medium, while the overexpression group was transfected with the FOXO3α-overexpressing lentivirus. After 48 h of transfection, complete medium was added to the cells in the overexpression group to establish a stably transfected cell line.

### Immunoprecipitation (co-IP)

THP-1 macrophages were lysed, and the IP lysate and protein samples were mixed with caspase-1 and caspase-3 antibodies and protein A/G-agarose beads (Absin, Shanghai, China) overnight at 4 ℃ with gentle shaking. Subsequent steps were performed according to the co-IP kit instructions. Negative controls using nonimmune IgG antibodies were used under the same experimental conditions to ensure the antibodies’ specificity for immunoprecipitation.

### Immunohistology of AS lesions

The heart’s aortic root and basal portion were fixed in 4% paraformaldehyde, followed by OCT compound embedding (Sakura, USA), before being cut into 6 μm-thick sections. Atherosclerotic lesions of the aortic root were observed by using HE staining. Oil red O staining was performed according to the manufacturer’s instructions to examine lipid deposition. Briefly, the frozen sections were soaked in reagent I for 10 min, followed by a 20-second wash using distilled water at 37 °C. Then, reagent II was added to the sections and incubated for 5 min, followed by a 60 s wash and microscopic examination. In addition, Oil red O staining, HE staining, Masson’s staining, and immunohistochemistry were performed on the aortic root. Images were captured using a confocal laser scanning microscope (NanoZoomerS210, Japan).

### Hoechst assay

Cells were fixed, washed twice with PBS and stained with Hoechst staining solution according to the manufacturer’s instructions (Beyotime Biotechnology, Jiangsu, China). Stained nuclei were observed under a fluorescence microscope (Nikon, Tokyo, Japan).

### Transmission electron microscopy (TEM)

After treatments, the cells were harvested by centrifugation and processed for TEM analysis on a JEM-1220 device (JEOL, Tokyo, Japan).

### Statistical analysis

All experiments were repeated at least three times independently. The data were analysed using one-way analysis of variance (ANOVA) and Student’s t test. The results are presented as the mean ± standard deviation (SD). A P value < 0.05 was considered statistically significant.

## Results

### Nicotine induces pyroptosis in THP-1 macrophages

**Fig. 1:**
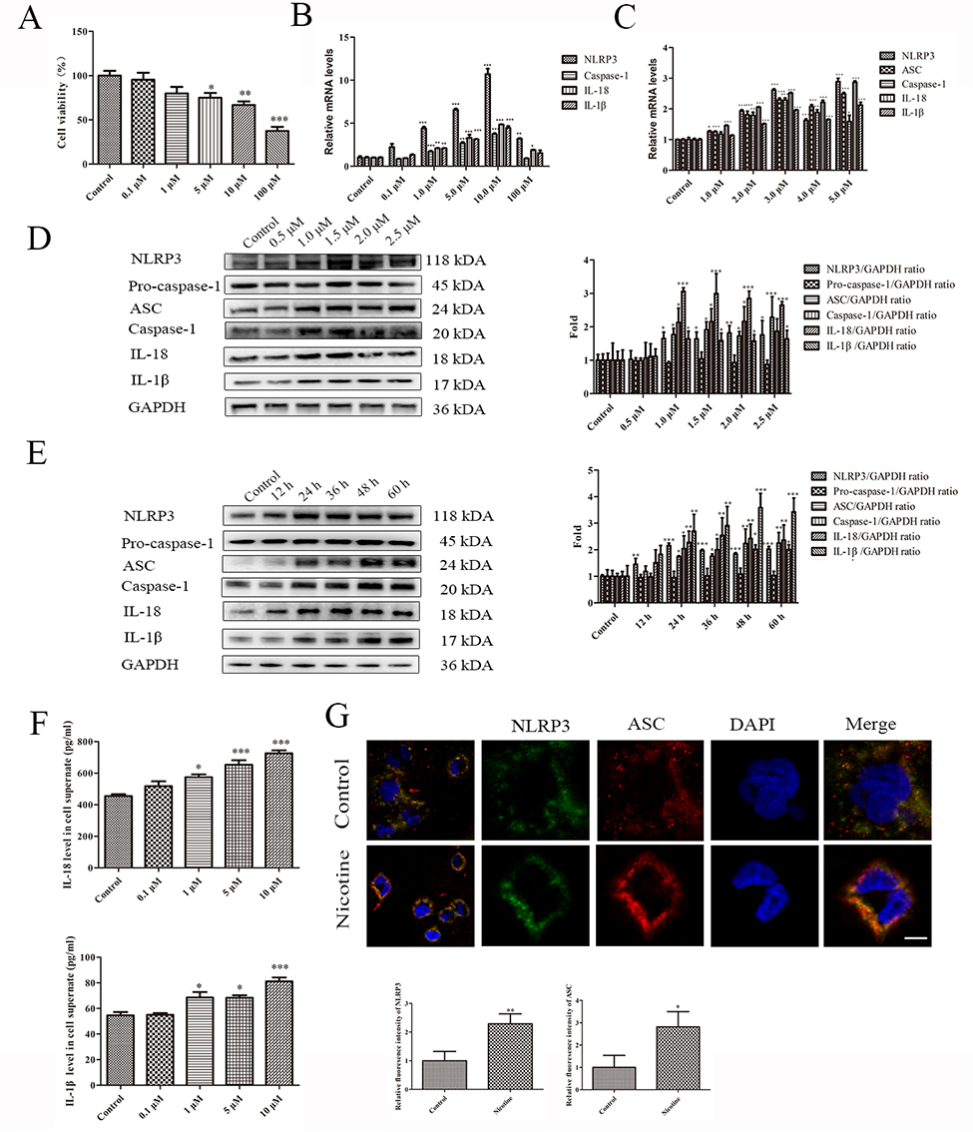
**Nicotine induces pyroptosis in THP-1 macrophages.** (**A**) Detection of cell viability (CCK-8 assay) after 24 h of exposure to different nicotine concentrations. (**B-C**) Dose-dependent mRNA expression of pyroptosis-related factors in untreated (control) and nicotine-treated macrophages (24 h exposure). (**D**) Dose-dependent expression of pyroptosis-related proteins in untreated (control) and nicotine-treated macrophages (24 h exposure). (**D**) Time-dependent expression of pyroptosis-related proteins in untreated (control) and nicotine-treated macrophages (1 µM). (**F**) ELISA results showing the secretion of IL-18 and IL-1β by macrophages treated with different nicotine concentrations for 24 h. (G) Effect of 1 µM nicotine on NLRP3 and ASC immunofluorescence in THP-1 macrophages (scale bar: 20 μm). Data are presented as mean ± SD (n = 3 represents three independent experiments per group); ^*^P < 0.05, ^**^P < 0.01, and ^***^P < 0.001 vs. control cells

The viability of nicotine-treated THP-1 macrophages was analyzed using the CCK-8 assay to determine the optimal pyroptosis induction conditions. Nicotine experiments revealed that macrophage viability was significantly reduced by exposure to ≥ 5 µM nicotine for 24 h (Fig. 1A). Consistently, the q-RT-PCR results confirmed that exposing macrophages to 1 µM nicotine for 24 h induced the expression of NLRP3, ASC, caspase-1 (caspase-1 represents cleaved-caspase-1), IL-18 (IL-18 represents cleaved-IL-18), IL-1β (IL-1β represents cleaved-IL-1β) (Fig. 1B-C). Similarly, the western blot results confirmed the q-RT-PCR results (Fig. 1D-E). Moreover, after incubation with 1 µM nicotine for 24 h, the ELISA results showed that IL-18 and IL-1β levels in cell culture supernatants increased significantly (Fig. 1F). During the activation process of NLRP3 inflammasome, ASC is activated to form polymers. In Fig. 1G, the aggregated ASC is observed under a microscope as characteristic ASC spots, a hallmark of NLRP3 inflammasome activation and pyroptosis.

### MT inhibits nicotine-induced pyroptosis in THP-1 macrophages

**Fig. 2:**
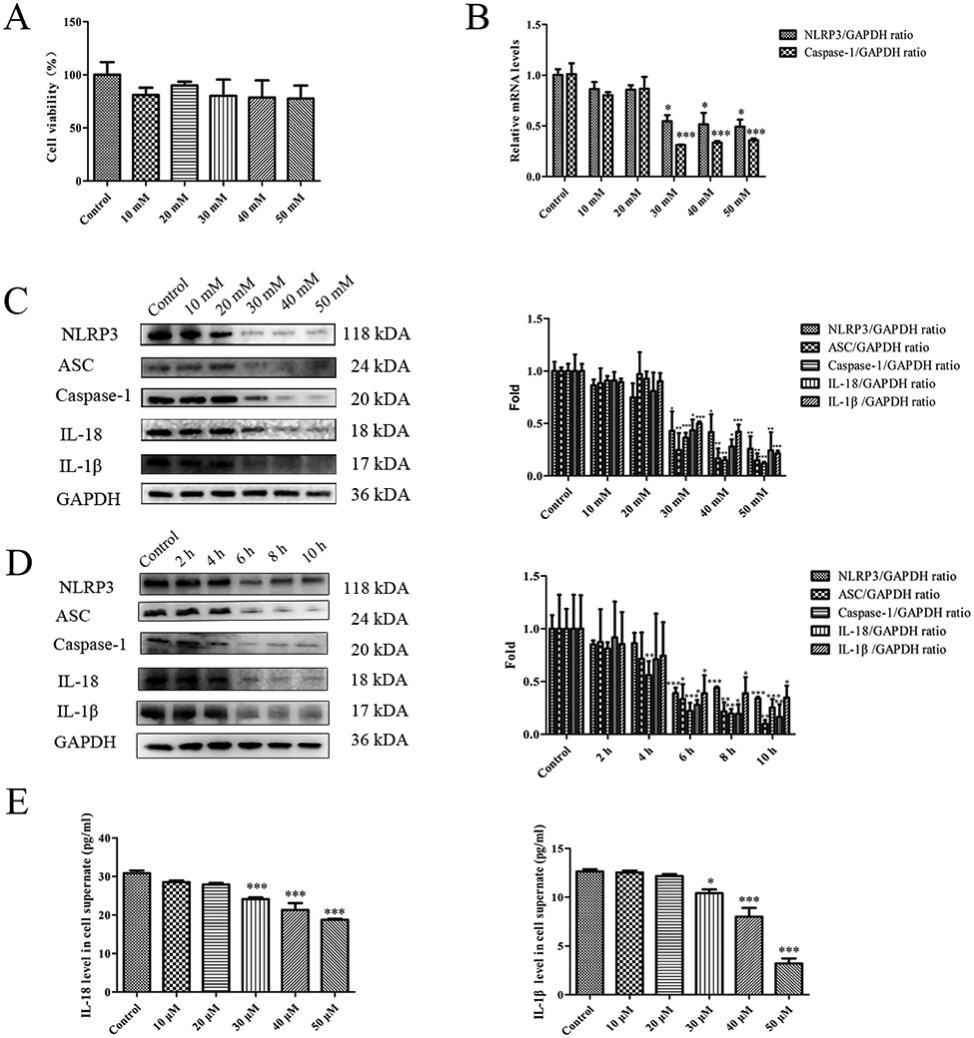
**MT inhibits nicotine-induced pyroptosis in THP-1 macrophages.** (**A**) Viability assay results of macrophages exposed to nicotine (1 µM, 24 h) and treated with different concentrations of MT (6 h). (**B**) The mRNA expression of NLRP3 and caspase-1 was analyzed by q-RT-PCR in macrophages (1 µ M nicotine, 24 h) without MT and macrophages (1 µ M nicotine, 24 h) treated with different concentrations of MT (6 h). (**C**) The pyroptosis-related protein expression was analyzed by WB in macrophages (1 µ M nicotine, 24 h) without MT and macrophages (1 µ M nicotine, 24 h) treated with different concentrations of MT (6 h). (**D**) The pyroptosis-related protein expression was analyzed by WB in macrophages (1 µ M nicotine, 24 h) without MT and macrophages (1 µ M nicotine, 24 h) treated with different incubation times of 30 mM MT. (**E**) The ELISA results showed that IL-18 and IL-1 β were secreted in the supernatant of macrophages (1 µ M nicotine, 24 h) without MT treatment or treated with different concentrations of MT (6 h). Data are presented as mean ± SD (n = 3 represents three independent experiments per group); ^*^P < 0.05, ^**^P < 0.01, and ^***^P < 0.001 vs. control cells

Cell viability assays revealed no significant changes in the survival rates of nicotine (1 µM, 24 h)-treated THP-1 macrophages when 0–50 mM MT was administered for 6 h (Fig. 2A ), which indicates that 0-50 m is a safe concentration for nicotine treated macrophages (1 µM nicotine, 24 h). As shown in Fig. 2B, C and D, THP-1 macrophages were administered 1 µM nicotine for 24 h which were treated with different concentrations of MT or without MT for 6 h. Finally, a concentration-dependent decrease in pyroptosis-related genes and proteins was observed using q-RT-PCR and WB. Furthermore, the ELISA results demonstrated a significant decrease in IL-18 and IL-1β levels in the supernatants of 1 µM nicotine-treated macrophages following 6 h of incubation with 30 mM MT, compared to the control group (macrophages treated with 1 µM nicotine for 24 h) (Fig. 2E). Therefore, a concentration of 30 mM and a 6 h exposure time were chosen as the optimal MT parameters to characterize the effects of MT on nicotine-induced pyroptosis in THP-1 macrophages.

### MT inhibits caspase-1-dependent pyroptosis in THP-1 macrophages induced by nicotine

**Fig. 3:**
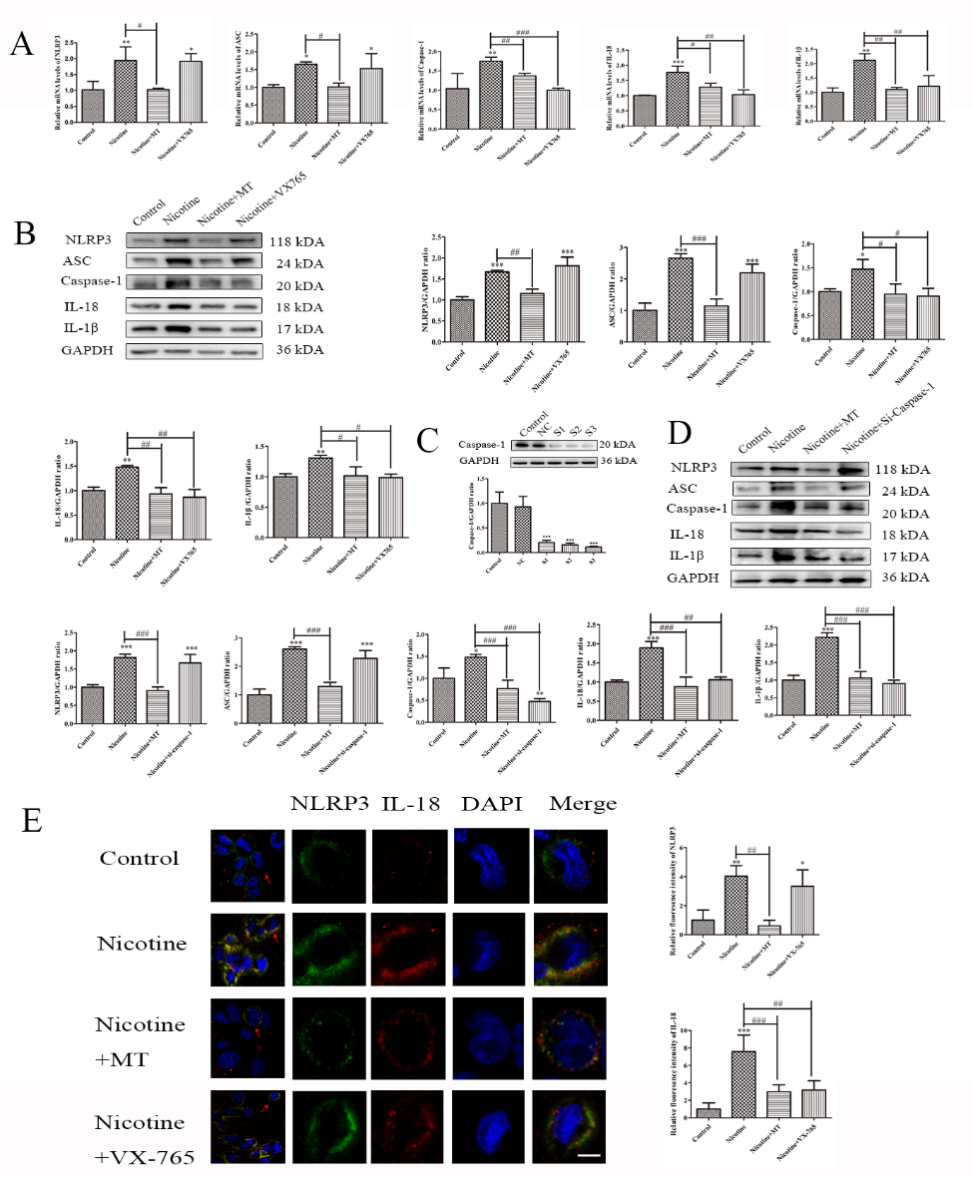
**MT inhibits caspase-1-dependent pyroptosis in THP-1 macrophages induced by nicotine.** (**A**) RT‒qPCR analysis of the effect of caspase-1 inhibition on pyroptosis-related mRNA expression. (**B**) WB analysis of the effect of caspase-1 inhibition on pyroptosis-related proteins. (**C**) Caspase-1 interference segment selection. (**D**) WB analysis of the effect of caspase-1 knockdown on pyroptosis-related proteins. (**E**) Effect of MT or VX-765 on NLRP3 and IL-18 immunofluorescence in nicotine-treated macrophages (scale bar: 20 μm). Data are presented as mean ± SD (n = 3 represents three independent experiments per group); ^*^P < 0.05, ^**^P < 0.01, and ^***^P < 0.001 vs. control cells. ^#^P < 0.05, ^##^P < 0.01, and ^###^P < 0.001 vs. nicotine-treated cells

We used VX-765, a potent and selective caspase-1 inhibitor, to analyze the expression of pyroptosis-related genes and proteins. The q-RT‒PCR and WB results showed that nicotine-induced caspase-1, IL-18, and IL-1β expression was significantly decreased by treatment with VX-765 (10 µM) or MT (30 mM) (Fig. 3A-B). To further examine the relationship between pyroptosis and caspase-1, we knocked down caspase-1 with siRNAs (Fig. 3C). Consistent with the previous results, IL-18 and IL-1β expression was reduced in nicotine-treated THP-1 macrophages following caspase-1 knockdown, while NLRP3 and ASC levels were unaffected (Fig. 3D). Furthermore, the immunofluorescence results were consistent (Fig. 3E), demonstrating that nicotine-induced inflammatory cell death depended on caspase-1.

### MT decreases pyroptosis in THP-1 macrophages by inhibiting ROS

**Fig. 4:**
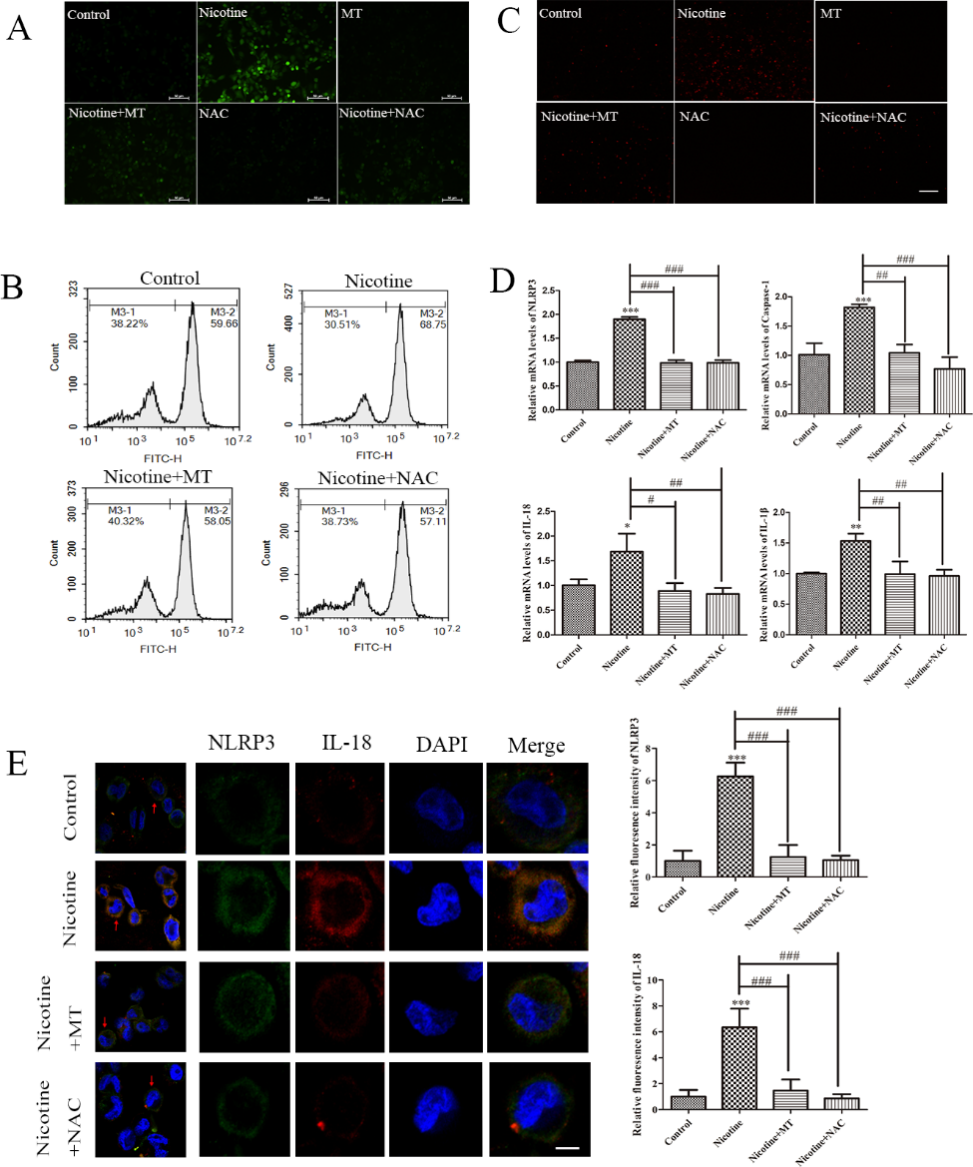
**MT inhibits ROS production in nicotine-treated THP-1 macrophages.** (**A**) DCFH-DA staining showing ROS generation in nicotine-treated macrophages exposed to NAC and MT (scale bar: 100 μm). (**B**) Flow cytometry showing ROS generation in nicotine-treated macrophages exposed to NAC and MT. (**C**) MitoSOX Red Mitochondrial Superoxide Indicator staining showing mitochondrion ROS generation in nicotine-treated macrophages exposed to NAC and MT (scale bar: 100 μm). (**D**) RT‒qPCR analysis of the effects of nicotine, NAC and MT on pyroptosis-related mRNA expression. (**E**) Effect of MT or NAC on NLRP3 and IL-18 immunofluorescence in nicotine-treated macrophages (scale bar: 20 μm). Data are presented as mean ± SD (n = 3 represents three independent experiments per group); ^*^P < 0.05, ^**^P < 0.01, and ^***^P < 0.001 vs. control cells. ^#^P < 0.05, ^##^P < 0.01, and ^###^P < 0.001 vs. nicotine-treated cells

Considering the detrimental effects of excessive inflammation and pyroptosis in AS, targeting mitochondrial ROS has emerged as a potential therapeutic approach. By inhibiting ROS production or scavenging ROS, we can potentially suppress the activation of the NLRP3 inflammasome and prevent pyroptosis [20]. To assess ROS generation during nicotine-induced pyroptosis and test whether MT could counteract this effect, NAC was used to inhibit ROS production in THP-1 macrophages. DCFH-DA staining showed that ROS levels induced by 1 µM nicotine were decreased by the addition of 30 mM MT, which was consistent with the results of NAC treatment (Fig. 4A). Moreover, ROS production was measured by DCFH-DA and flow cytometry and showed that ROS generation in nicotine-treated THP-1 macrophages was decreased by treatment with NAC or MT (Fig. 4B). We further detected mitochondrial ROS using MitoSOX Red Mitochondrial Superoxide Indicator staining, and the results showed a consistent trend between mitochondrial ROS and intracellular ROS changes (Fig. 4C). Furthermore, the q-RT‒PCR results further confirmed that NAC could inhibit pyroptosis-related mRNA expression (Fig. 4D). The immunofluorescence results showed that NAC or MT inhibited the nicotine-induced expression of the pyroptosis-related proteins NLRP3 and IL-18 (Fig. 4E). The above results reveal that MT inhibits nicotine-induced macrophage pyroptosis by inhibiting the number of ROS in mitochondria.

### MT inhibits nicotine-induced macrophage pyroptosis via the SIRT3/FOXO3α/ROS axis

**Fig. 5:**
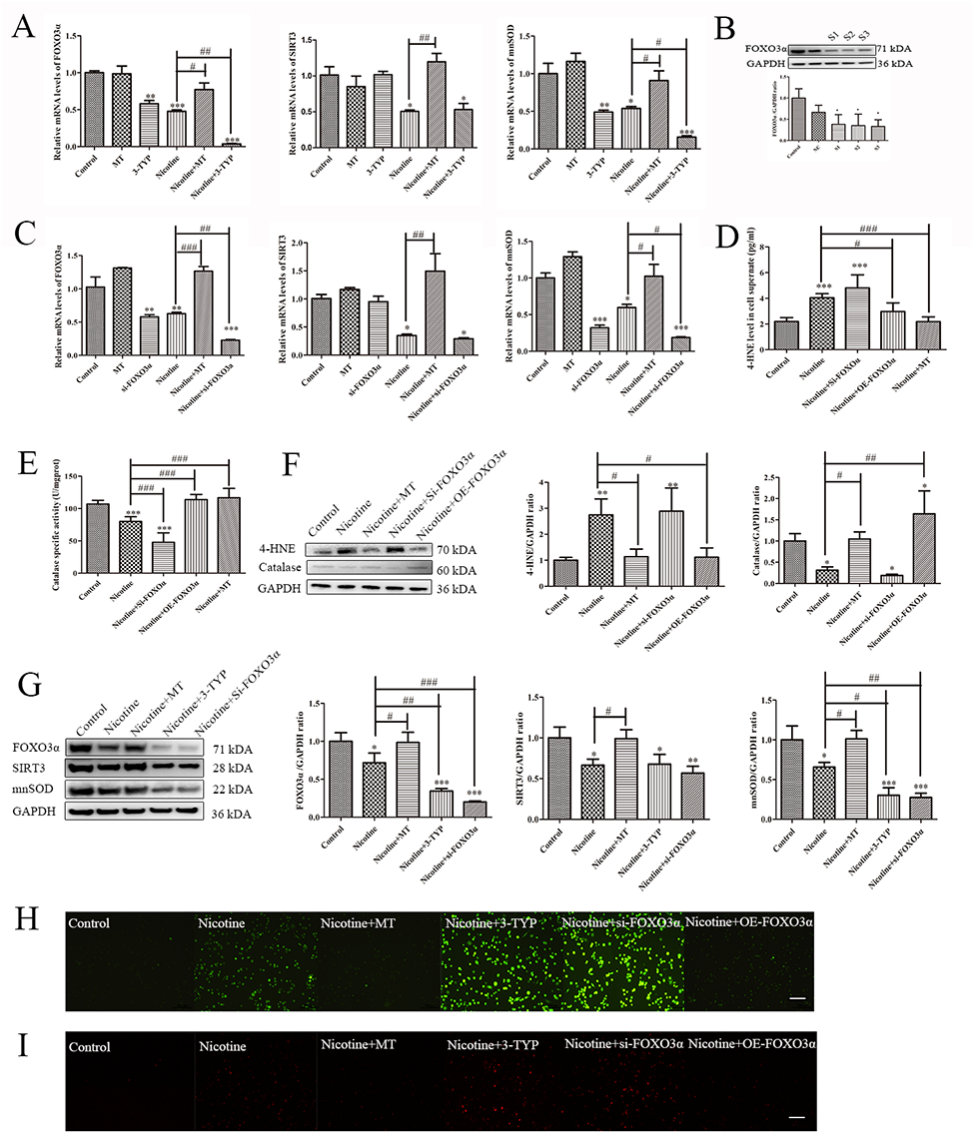
**MT inhibits pyroptosis through the SIRT3/FOXO3α/ROS axis.** (**A**) q-RT‒PCR analysis of FOXO3α, SIRT3 and mnSOD after the different treatments. (**B**) FOXO3α interference segment selection. (**C**) q-RT‒PCR analysis of FOXO3α, SIRT3 and mnSOD after the different treatments. (**D**) ELISA determination of 4-HNE secretion. (**E**) Quantification of catalase activity (colorimetric detection). (**F**) WB analysis of the expression of 4-HNE and catalase in macrophages. (**G**) WB analysis of the protein expression of FOXO3α, SIRT3, and mnSOD in macrophages. (**H**) DCFH-DA staining to detect ROS levels in cells after the different treatments (scale bar: 100 μm). (**I**) MitoSOX Red Mitochondrial Superoxide Indicator staining to detect mitochondrial ROS levels after the different treatments (scale bar: 100 μm). Data are presented as mean ± SD (n = 3 represents three independent experiments per group); ^*^P < 0.05, ^**^P < 0.01, and ^***^P < 0.001 vs. control cells. ^#^P < 0.05, ^##^P < 0.01, and ^###^P < 0.001 vs. nicotine-treated cells

As shown in Fig. 5A-C, the PCR results showed that the order of the three was SIRT3-FOXO3α-mnSOD. FOXO3α expression was downregulated by SIRT3 inhibition (3-TYPE is a SIRT3 activity inhibitor, but does not affect the expression of SIRT3.), while FOXO3α interference did not affect SIRT3 expression, indicating that SIRT3 regulates oxidative stress and pyroptosis upstream of FOXO3α. Next, we overexpressed or interfered with FOXO3α expression and detected the levels of 4-hydroxynonenal (4-HNE) protein adducts, indicative of lipid peroxidation, and a critical antioxidant enzyme, i.e., catalase. Nicotine or si-FOXO3α intervention significantly increased oxidative stress levels, while OE-FOXO3α or MT treatment attenuated this effect (Fig. 5D-F). To further confirm the upstream and downstream relationship at the protein level of FOXO3α, SIRT3, and mnSOD, we added SIRT3 inhibitor 3-TYP and interference with FOXO3α to nicotine-induced macrophages. After detecting the expression changes of three proteins, it was found that they were consistent with the gene level (Fig. 5G). As shown in Fig. 5H-I, intracellular and mitochondrial ROS levels were detected by DCFH-DA and MitoSOX Red Mitochondrial Superoxide Indicator staining after the different treatments to determine further the upstream and downstream relationship between the three factors. These findings indicate that SIRT3 and FOXO3α coregulate oxidative stress, and SIRT3 is located upstream of FOXO3α. Furthermore, we showed that MT treatment could alleviate nicotine-induced macrophage pyroptosis through the SIRT3/FOXO3α/ROS axis.

### Apoptosis and pyroptosis coregulate MT-treated macrophages

**Fig. 6:**
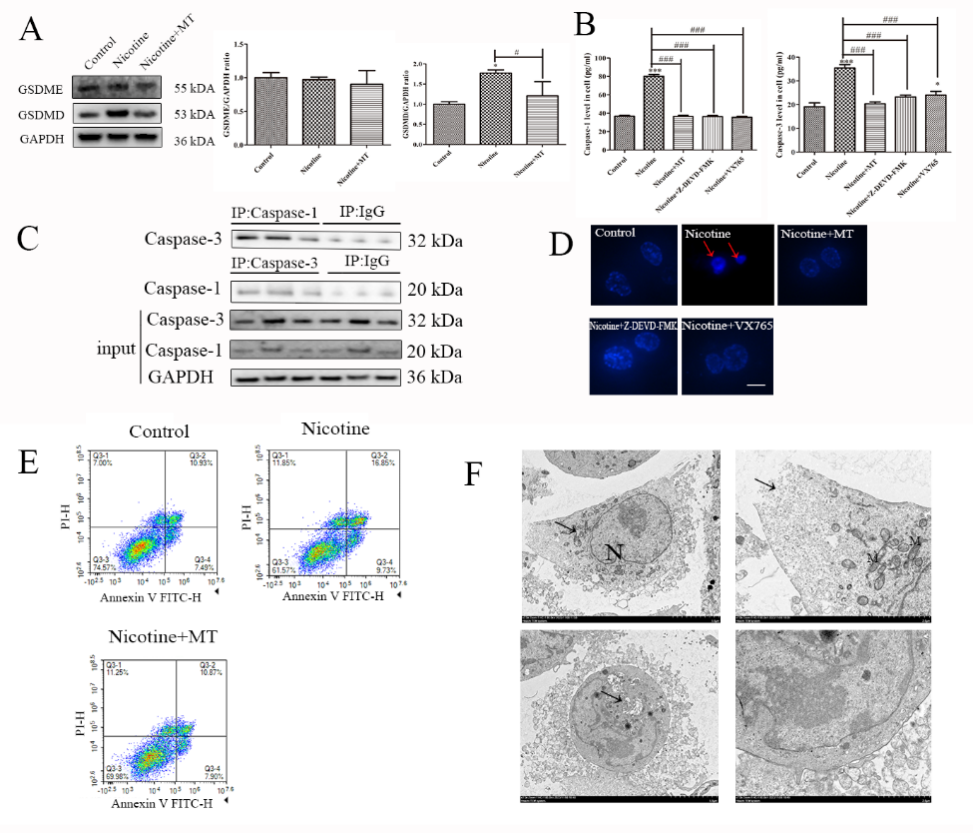
**MT induces the interaction of caspase-1 and caspase-3 in nicotine-treated macrophages.** (**A**) The WB results showed the expression of GSDME and GSDME in macrophages under different treatments, and the specific pathway of pyroptosis has been identified. (**B**) ELISA results showing macrophage secretion of caspase-1 and caspase-3 after the different treatments. (**C**) Representative western blots showing caspase-3 expression following immunoprecipitation with an anti-caspase-1 antibody (top), caspase-1 expression following immunoprecipitation with an anti-caspase-3 antibody (middle), and caspase-1 and caspase-3 expression in total cell lysates (bottom). (**D**) Hoechst staining of THP-1 macrophages (When apoptosis occurs, the nucleus condenses. Indicate with a red arrow.) (scale bar: 20 μm). (**E**) Flow cytometry was used to detect apoptosis in macrophages after the different treatments. (F) TEM analysis of ultrastructural alterations in 1 µM THP-1 macrophages (scale bar: 2 μm). Data are presented as mean ± SD (n = 3 represents three independent experiments per group); ^*^P < 0.05, ^**^P < 0.01, and ^***^P < 0.001 vs. control cells. ^#^P < 0.05, ^##^P < 0.01, and ^###^P < 0.001 vs. nicotine-treated cells

Apoptosis is different from apoptosis: apoptosis mainly relies on the activity of caspase-3, leading to slow cell death [30]; Apoptosis is mediated by caspase-1, which relies on the activation of inflammasomes and promotes rapid cell death and inflammatory response. On the other hand, during the process of cell apoptosis, the activated Caspase-3 protein cleaves GSDME into GSDME-C and GSDME-N fragments, where the GSDME-N fragment mediates the progression of pyroptosis [15]. In order to further determine the specific pathway of nicotine-induced macrophage pyroptosis, we detected the expression of GSDME and GSDMD in macrophages after different treatments (Fig. 6A). WB results showed that the nicotine-induced pyroptosis pathway is a classic pyroptosis pathway regulated by caspase-1-GSDMD, and caspase-3 only activated apoptosis in this study. Further, we examined whether there was a specific interaction between caspase-1 and caspase-3, which are members of the same family, that leads to the same progression and regression of pyroptosis and apoptosis. As shown in Fig. 6B, the ELISA results showed that caspase-3 inhibitors Z-DEVD-FMK inhibited the expression of caspase-1, and caspase-1 inhibitors VX765 inhibited the expression caspase-3. In THP-1 macrophages treated with nicotine, caspase-3 levels were increased, according to coimmunoprecipitation results. In addition, caspase-1 was precipitated after adding caspase-3 antibodies and vice versa (Fig. 6C), confirming an interaction between caspase-1 and caspase-3. The interaction between caspase-1 and caspase-3 shows that caspase-3 is abundantly expressed when the nicotine-induced increase in caspase-1 in macrophages promotes the progression of pyroptosis. Finally, we used Hoechst staining and flow cytometry to detect the expression changes of apoptosis after different treatments. Hoechst staining of THP-1 macrophages treated with nicotine revved condensed bright nucleus typical of apoptotic dead cells. However, adding MT, Z-DEVD-FMK, and VX765 reversed this phenomenon (Fig. 6D).

Moreover, apoptosis levels after the various treatments were detected by flow cytometry, as shown in Fig. 6E. They used Annexin V-FITC labeled fluorescence to identify early cell apoptosis and labeled necrotic cells with propidium iodide (PI). We found that nicotine induces macrophage apoptosis, and MT reverses this phenomenon. In conclusion, the data from Fig. 6A-E show that MT inhibits nicotine-induced pyroptosis, which involves apoptosis. Figure 6 F shows 1 µM nicotine-induced morphological changes in macrophages. The nucleus (N) is nearly circular, with pyknosis, widening of the local perinuclear space (at the arrow), moderate swelling of mitochondria (M), and disruption of cell membrane integrity (at the arrow), all of which are manifestations of macrophage pyroptosis. Cells become smaller and rounder, with suspected chromatin-like structures near the nuclear membrane, the cytoplasm has more vacuolar structures (arrows), and the cell membrane is intact, all manifestations of apoptosis. The TEM results reveal 1 µM nicotine-induced pyroptosis and apoptosis in THP-1 macrophages.

### MT alleviates nicotine-induced AS

**Fig. 7:**
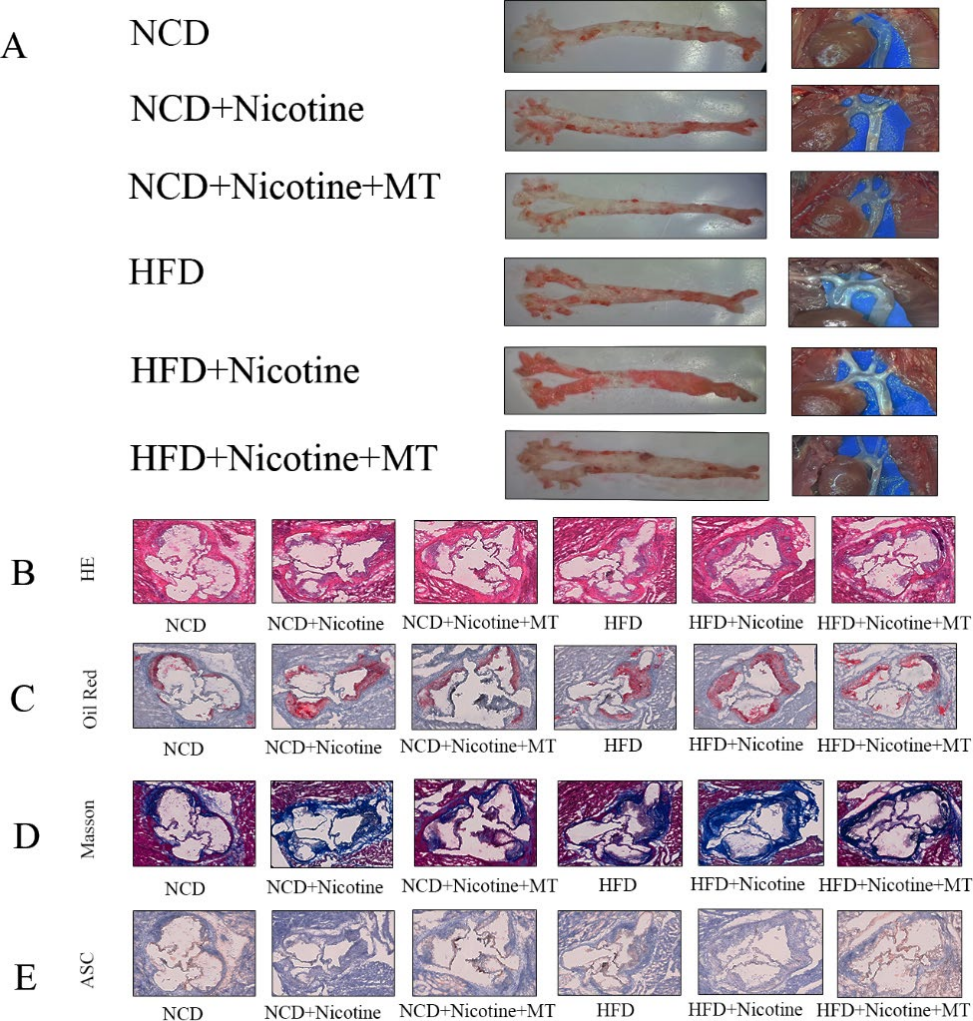
**MT alleviates AS.** (**A**) Representative images of en face samples stained with Oil Red O and in vivo photos of the aortas of ApoE^−/−^ mice in the NCD, NCD + nicotine, NCD + nicotine + MT, HFD, HFD + nicotine, and HFD + nicotine + MT groups. (**B**) HE staining of aortic root sections shows atherosclerotic lesions. (**C**) Oil red O staining of aortic root sections shows lipid deposition. (**D**) Masson staining was used to determine the content of collagen fibers in the root of the aortic arch (Blue represents collagen fibers.). (**E**) The expression of ASC was detected by immunohistochemistry (Brown represents ASC.). n = 10 mice in each group. The scale bar is 600 μm. ^*^P < 0.05, ^**^P < 0.01, and ^***^P < 0.001 vs. NCD. ^#^P < 0.05, ^##^P < 0.01, and ^###^P < 0.001 vs. HFD

The oil red O and HE staining indicated severe AS was successfully induced in the ND + nicotine, HFD, and HFD + nicotine groups. In addition, there was a significant increase in lesion size and lipid content in the ND + nicotine, HFD, and HFD + nicotine groups compared to the ND group. Interestingly, under nicotine and HFD conditions, mice treated with MT exhibited significant reductions in AS lesion areas. In contrast, mice fed a standard diet showed no significant changes (Fig. 7A-C). Based on these results, MT can improve AS lesions. Next, the root of the aortic arch was subjected to Masson staining to determine the collagen fiber content. Modified Masson staining showed blue collagen fibers, red muscle fibers, cytoplasm, cellulose, keratin, red blood cells, and blue-brown nuclei. The results showed that compared with the control group and MT treatment, the proportion of collagen fibers in the aortic root section of mice fed with nicotine and a high-fat diet was higher (Fig. 7D). The immunohistochemical results revealed that ASC, a pyroptosis-related protein, was significantly increased in the groups that drank nicotine water solution and were fed a high-fat diet. ASC expression was decreased after MT addition (Fig. 7E). Long-term administration of MT can cause changes in hormone levels. To ensure the safety of the therapeutic concentration, we measured testosterone levels in mouse serum. We found no significant change after MT treatment (Supplementary Fig. 1), ensuring the safety of our experiment.

### OX-LDL accelerates nicotine-induced pyroptosis

**Fig. 8:**
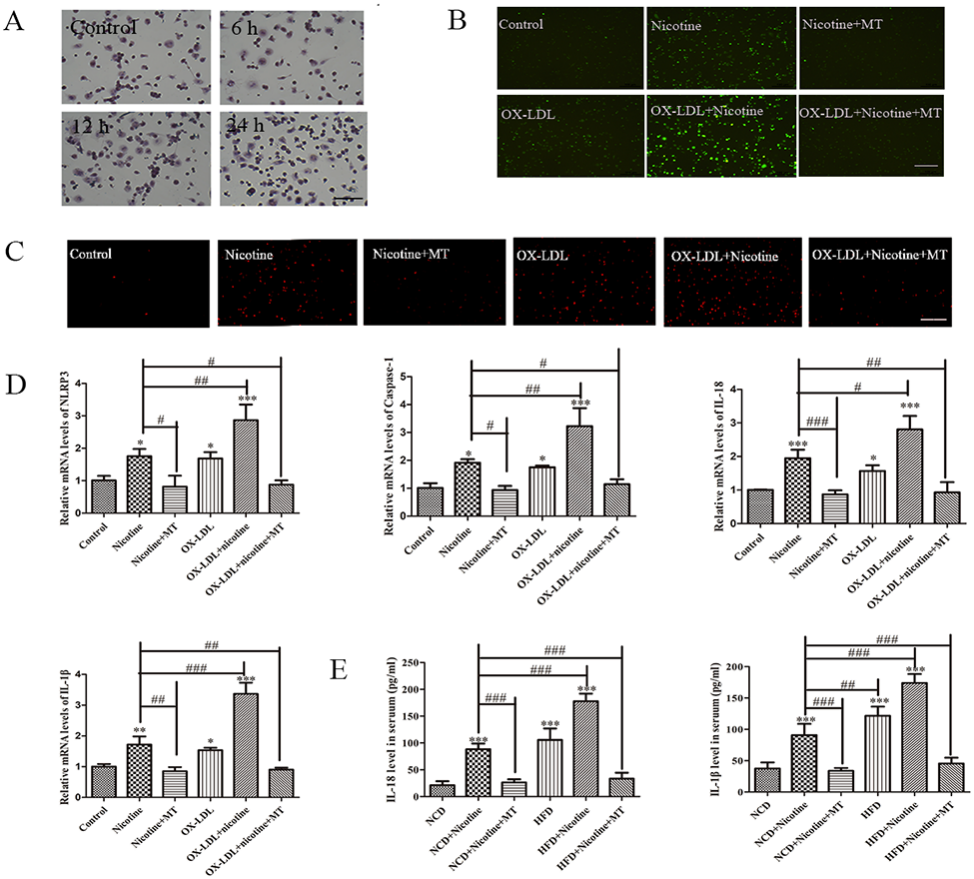
**MT inhibits nicotine-induced pyroptosis in foam cells.** (**A**) Representative images of Oil Red O-stained THP-1 macrophages following incubation with ox-LDL for 6, 12, 24, and 48 h (scale bar: 50 μm). (**B**) DCFH-DA staining to detect ROS levels in cells after the different treatments. (**C**) MitoSOX Red Mitochondrial Superoxide Indicator staining to detect ROS levels in mitochondrion after the different treatments. (**D**) q-RT-PCR analysis of changes in the expression of macrophage pyroptosis-related genes after the different treatments. (**E**) ELISA results showing the secretion of IL-18 and IL-1β in the serum of mice with different treatments. Data are presented as mean ± SD (n = 3 represents three independent experiments per group); ^*^P < 0.05, ^**^P < 0.01, and ^***^P < 0.001 vs. control cells. ^#^P < 0.05, ^##^P < 0.01, and ^###^P < 0.001 vs. nicotine-treated cells. n=10 mice in each group. ^*^P < 0.05, ^**^P < 0.01, and ^***^P < 0.001 vs. NCD. ^#^P < 0.05, ^##^P < 0.01, and ^###^P < 0.001 vs. HFD

As shown in Fig. 8A, oil red O staining was used to detect the effect of ox-LDL on macrophages at different time points, and it was found that ox-LDL could induce the foaming of macrophages after 12 h. The results of DACH-DA/MitoSOX Red Mitochondrial Superoxide Indicator staining showed that ox-LDL accelerated the generation of ROS induced by nicotine (Fig. 8B-C). Importantly, the q-RT-PCR results demonstrated that OX-LDL could aggravate nicotine-induced macrophage pyroptosis (Fig. 8D). We detected changes in the levels of IL-18 and IL-1β in the serum of mice after the different treatments and found that the results were consistent with the mRNA results (Fig. 8E). This finding suggests that high-fat diet feeding may accelerate atherosclerotic plaque accumulation by causing pyroptosis in macrophages.

## Discussion

Previous studies have described the role of MT in inhibiting pyroptosis, and several possible pathways have been confirmed [31–33]. However, in this study, we identified a novel mechanism by which MT alleviates AS by cutting off the occurrence of oxidative stress and inhibiting macrophage pyroptosis. Our findings provided an experimental basis for the clinical application of MT in AS management and suggested the great potential of the SIRT3/FOXO3α/ROS axis as a target for AS treatment.

Our research results provide an experimental basis for the clinical application of MT in treating AS and suggest the presence of a signal “crosstalk” between different PCDs. It is critical to put molecular mechanisms of mutual interference between different PCDs into the context of tissue homeostasis and pathology in CVD. While remaining significantly different from pyroptosis and apoptosis in many ways, there is co-existence and mutual crosstalk among them. Indeed, kinds of PCDs constitute a pluralistic, coordinated cell death system in which one pathway can flexibly compensate for the other. Studying the molecular mechanisms of these two cell death modes and identifying the relationships and regulatory commonalities between pyroptosis and apoptosis is of great significance for studying the pathogenesis and prevention of CVD.

Typically, we think fat and cholesterol are essential factors that cause AS. However, in this study, we excluded the interference of fat and cholesterol on ApoE^−/−^ mouse models. We confirmed that nicotine treatment alone could accelerate plaque formation through inflammatory responses, while MT could exert an anti-inflammatory effect. Moreover, the effect of OX-LDL on promoting pyroptosis was further investigated in vitro. Taken together, our findings confirmed the stimulatory effects of nicotine and OX-LDL on the inflammatory response and formation of atherosclerotic plaques, as well as the therapeutic effect of MT.

As is well known, smoking exacerbates age-related diseases such as CVD. Smoking increases free cholesterol, ceramides, and phospholipids in the plasma, free and esterified cholesterol in the liver, and cholesterol esters and lysophosphatidylcholine in the aorta. Interestingly, Kunitomo et al. demonstrated that ApoE^−/−^ mice exposed to smoke in the gas phase (without nicotine and tar) induced oxidative stress in the blood circulation and aorta while increasing serum total cholesterol [34]. These data indicate that in addition to nicotine, other components in cigarette smoke also contribute to the development of CVD. People used to mainly focus on the impact of cigarette smoke on animal models, but now they are shifting their attention to the main substances in cigarettes, such as nicotine. Nicotine, as the primary harmful substance in tobacco, plays an essential role in the occurrence and development of CVD, and nicotine exposure is inevitable in daily life. Nicotine exposure causes cardiovascular diseases, leading to myocardial damage, remodeling, fibrosis, atrial fibrillation, arrhythmia, and more [35]. With the increasing number of smokers, the harm of nicotine to humans is becoming increasingly significant. Therefore, it is crucial to clarify the cardiotoxicity of nicotine. The mouse model is most commonly used for nicotine research [36], where mice use an enzyme homologous to humans to metabolize nicotine and form a large amount of cotinine. In this study, we used a mouse model to administer nicotine through drinking water to better investigate the impact of nicotine as an independent factor on cardiovascular health.

MT is an endocrine hormone that regulates the synthesis and secretion of nearly all hormones. Numerous studies have demonstrated that the effect of MT on reproduction is mediated by the hypothalamus-pituitary-gonadal axis and that exogenous MT reduces the testicular quality of rats [37, 38]. To verify whether our preliminary experimental MT treatment concentration could affect testosterone levels in male mice, ELISA kits were used to detect testosterone levels in mouse serum (Supplementary Fig. 1). The results revealed that the MT concentrations used had no discernible effect on testosterone levels, indicating that the therapeutic concentration that was chosen was safe. There are two types of receptors for MT binding: MT1 and MT2. Previous studies have found the presence of these two melatonin receptors in the cardiovascular system [39]. The MT1 receptor plays a vital role in controlling rhythm, and the MT2 receptor is closely related to the cyclic activity of MT in the body. Previous studies have found that changes in MT receptor activity can cause mice (C57BL/6J) to choose nicotine [40], but it is still unclear whether nicotine can cause macrophage inflammation by altering MT receptor activity. This mechanism may also exist in our research and needs further verification.

As a component of cigarettes, nicotine is an addictive substance and has been linked to cell apoptosis [41]. With increasing interest in pyroptosis in basic research, a link has gradually been established between nicotine and pyroptosis. We observed significant changes in apoptosis levels after successfully establishing a nicotine-induced pyroptosis macrophage model. Caspase-3 acts as the executor of apoptosis, while caspase-1 is not thought to be directly involved in the transduction of apoptotic signals and is mainly involved in the activation of interleukin precursors. Han [42] et al. found that Caspase-1-positive cells were significantly increased 12 h after ischaemia‒reperfusion injury. Similarly, caspase-3-positive cells were significantly increased 12 h after injury and resolved after spinal cord ischaemia‒reperfusion injury. During neuronal apoptosis, both caspase-1 and caspase-3 are involved in injury regulation. Therefore, we surmised the presence of crosstalk between caspase-1 and caspase-3. Importantly, our co-IP experiment confirmed this hypothesis; however, the intricate mechanism remains unclear because cell death is a complex process that requires further study.

During the typical inflammasome-induced pyroptosis process, GSDMD is hydrolyzed and cleaved by activated caspase-1 protein, producing active fragments with membrane pore formation ability, thereby secreting and releasing IL-1 β and IL-18 [43]. In addition, caspase-3 activates GSDME by cleaving Asp270, which can also transform relatively slow non-inflammatory apoptosis into faster inflammatory pyroptosis [43]. Previous studies reported that GSDME and GSDMD could induce the transition from apoptosis to pyroptosis [14, 15]. Our study found that nicotine-induced macrophage pyroptosis did not involve GSDME, and only GSDMD was involved in this process. Therefore, in this study, caspase-3 only participated in apoptosis and did not participate in pyroptosis. In addition, we discovered that not only can apoptosis promote the occurrence of pyroptosis, but the two processes could work synergistically. Both mechanisms were increased in nicotine-treated macrophages and were reduced following MT treatment. A certain level of apoptosis is protective by eliminating senescent cells, whereas excessive apoptosis can result in immune silencing and promote the inflammatory response [43, 44]. Uncertainty exists about whether apoptosis induced by pyroptosis protects cells from the pyroptosis-induced inflammatory response or further accelerates the inflammatory response of macrophages; therefore, additional research is needed. On the other hand, controlling the degree of apoptosis during pyroptosis may protect cells from inflammatory damage and slow the progression of AS. Therefore, an in-depth understanding of the interactions and intermediate molecular mechanisms between the various modes of macrophage death can provide new directions and strategies for treating CVD-related diseases.

## Conclusion

**Fig. 9:**
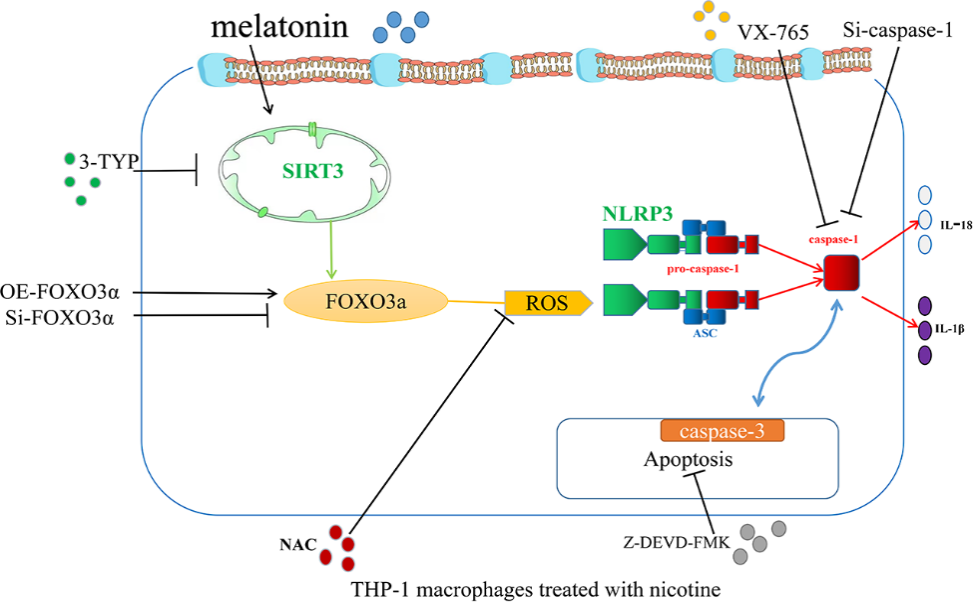
Schematic representation of the effects of MT on nicotine-treated THP-1 macrophages. Nicotine exposure induces ROS production, which promotes NLRP3 inflammasome formation, leading to the activation of caspase-1. Activated caspase-1 triggers pyroptosis through membrane pore formation, DNA fragmentation, and the release of mature IL-1β and IL-18. The ensuing sterile inflammation contributes to the progression of AS. Moreover, MT normalizes Sirt3 expression and promotes FOXO3α expression in nicotine-treated macrophages. This reduces ROS production and prevents oxidative stress, which inhibits NLRP3 inflammasome formation and prevents pyroptosis. The process by which MT inhibits nicotine-induced pyroptosis also involves apoptosis, which is mainly due to the interaction between caspase-1 and caspase-3. ↑ induce; ⊥ inhibit

Herein, we focused on inhibiting the activation of NLRP3 upstream of pyroptosis by reducing ROS levels and discovered the specific molecular mechanism: the SIRT3/FOXO3α/ROS axis (Fig. 9). In addition, this study showed that pyroptosis and apoptosis communicated through the interaction of caspase-1 and caspase-3 (Fig. 9). Collectively, our findings provide a new target for inhibiting inflammation to treat AS.

### Electronic supplementary material

Below is the link to the electronic supplementary material.

Supplementary Material 1

Supplementary Material 2

Supplementary Material 3

Supplementary Material 4

## Data Availability

All data generated or analyzed during this study are included in this published article.
