# Supplementary figures and images for: Melatonin alleviates pyroptosis by regulating the SIRT3/FOXO3α/ROS axis and interacting with apoptosis in Atherosclerosis progression
Source: Biol Res. 2023 Dec 2;56:62. doi: 10.1186/s40659-023-00479-6 (PMC10693060; doi:10.1186/s40659-023-00479-6)

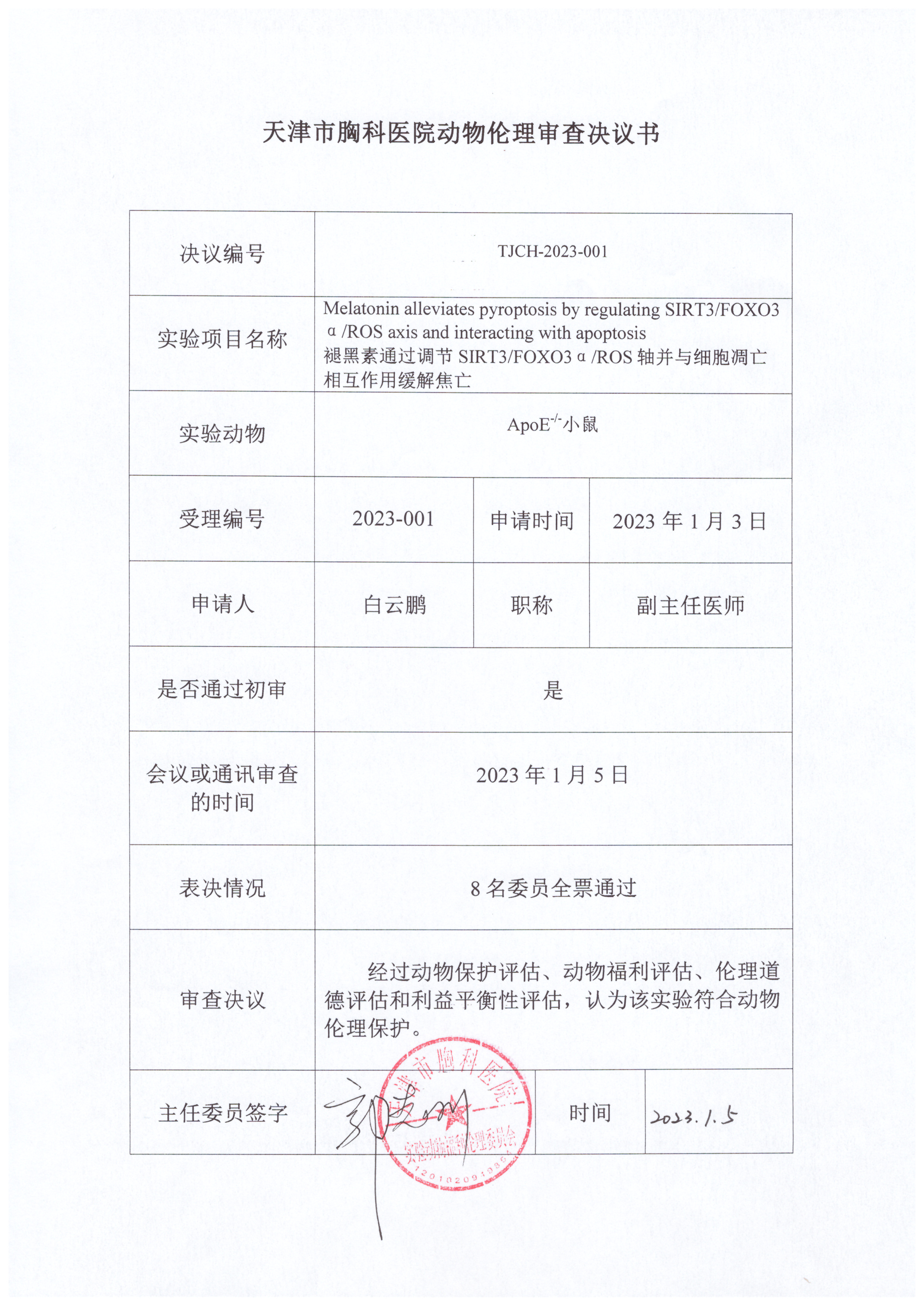

Supplement: Supplementary file 1 — Supplementary Material 1 [file 40659_2023_479_MOESM1_ESM.jpg]

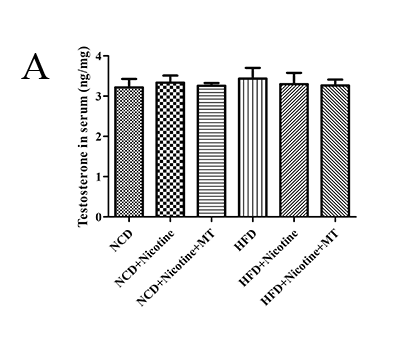

Supplement: Supplementary file 2 — Supplementary Material 2 [file 40659_2023_479_MOESM2_ESM.png]
