# Supplementary material for: Melatonin alleviates pyroptosis by regulating the SIRT3/FOXO3α/ROS axis and interacting with apoptosis in Atherosclerosis progression
Source: Biol Res. 2023 Dec 2;56:62. doi: 10.1186/s40659-023-00479-6 (PMC10693060; doi:10.1186/s40659-023-00479-6)
Supplement: Supplementary file 3 — Supplementary Material 3 [file 40659_2023_479_MOESM3_ESM.docx]

**Supplementary Figure 1.** (A) ELISA results show secretion of testosterone in the serum of mice with different treatments. n=10 mice in each group. ^*^P<0.05, ^**^P<0.01, and ^***^P<0.001 vs. NCD. ^#^P<0.05, ^##^P<0.01, and ^###^P<0.001 vs. HFD.
