# Supplementary material for: Melatonin alleviates pyroptosis by regulating the SIRT3/FOXO3α/ROS axis and interacting with apoptosis in Atherosclerosis progression
Source: Biol Res. 2023 Dec 2;56:62. doi: 10.1186/s40659-023-00479-6 (PMC10693060; doi:10.1186/s40659-023-00479-6)

Explanation: Due to the need to display the results of proteins with different molecular weights on the same PVDF membrane, we performed imprinting cleavage before antibody hybridization. To ensure the rigor of the experiment, we cut the blots of the exact result graph of the same grouping on a PVDF membrane. However, the exposure time of different bands is inconsistent due to differences in antibodies. Therefore, we adopted a separate color development method (simultaneous color development may lead to underexposure/overexposure of some blots).

1D-NLRP3

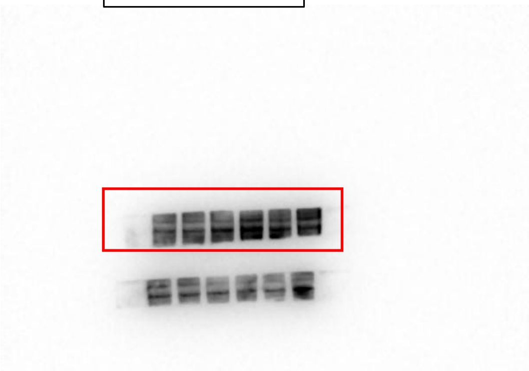

1D-Pro-caspase-1

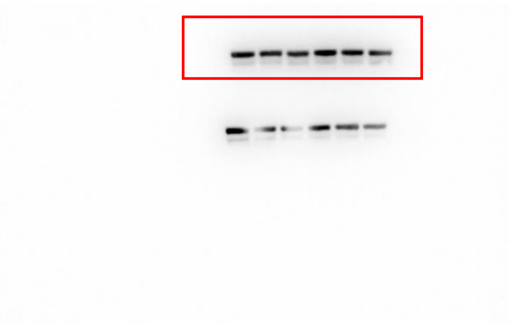

1D-ASC

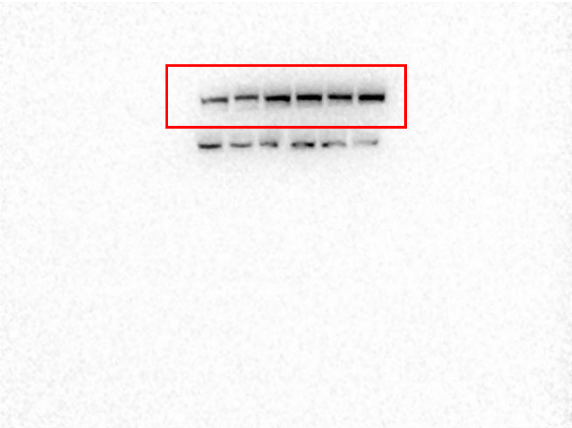

1D-Caspase-1

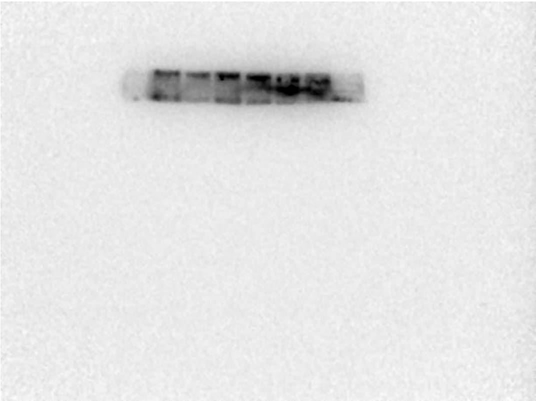

1D-IL-18

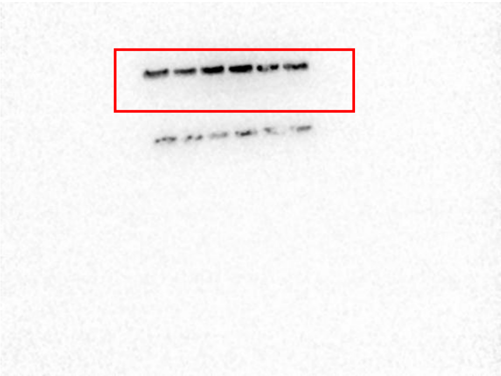

1D-IL-1β

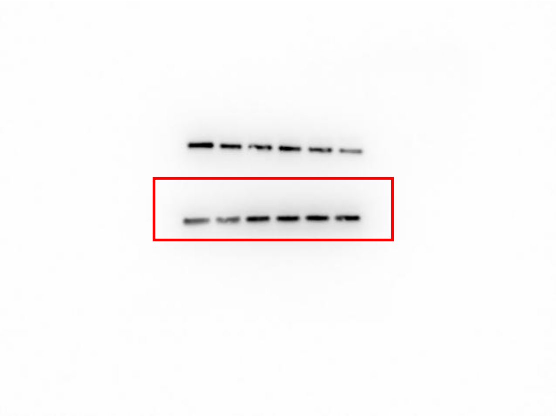

1D-GAPDH

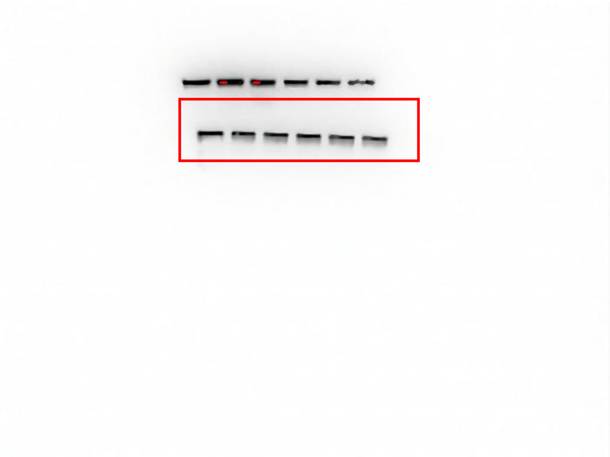

1E-NLRP3

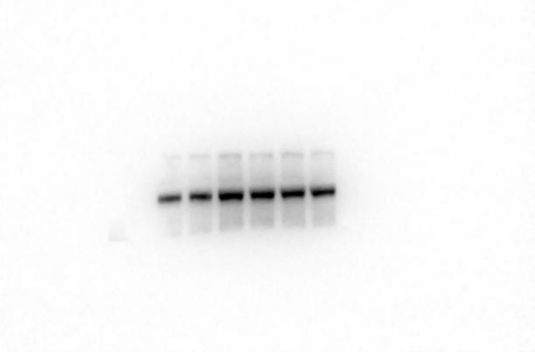

1E-Pro-caspase-1

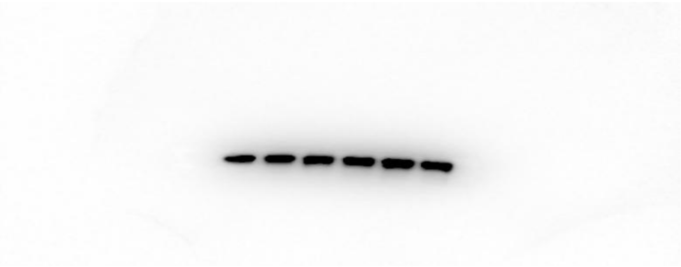

1E-ASC

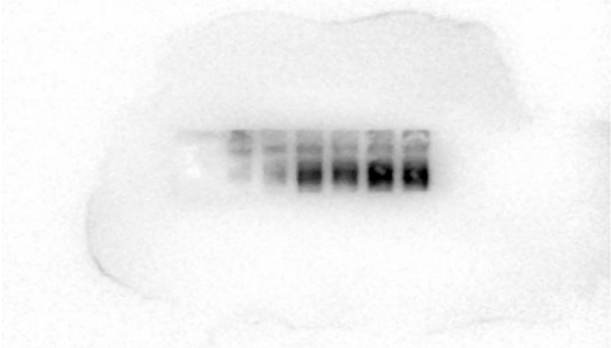

1E-Caspase-1

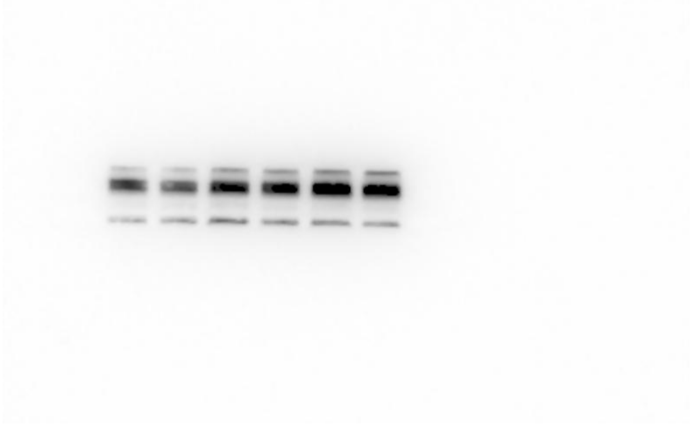

1E-IL-18

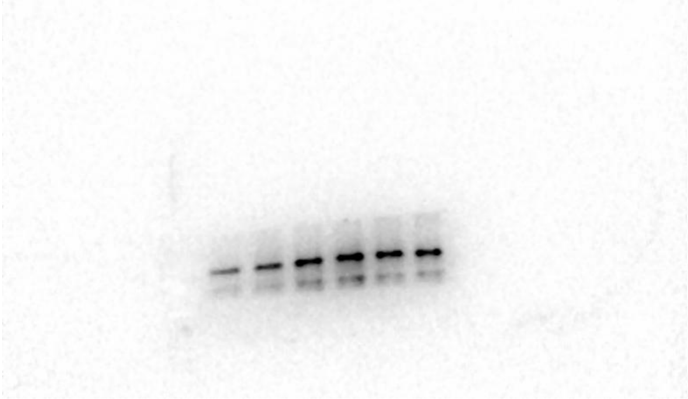

1E-IL-1 β

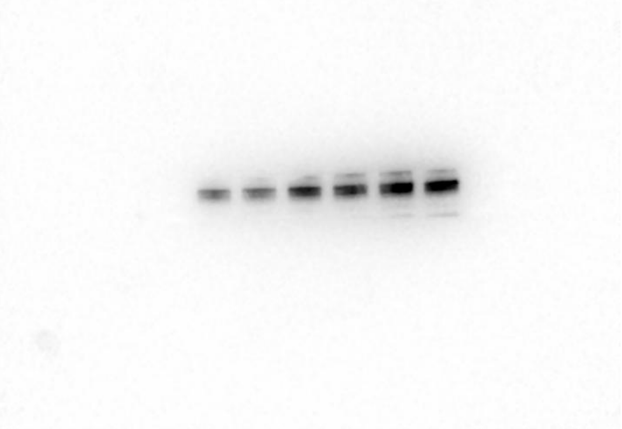

1E-GAPDH

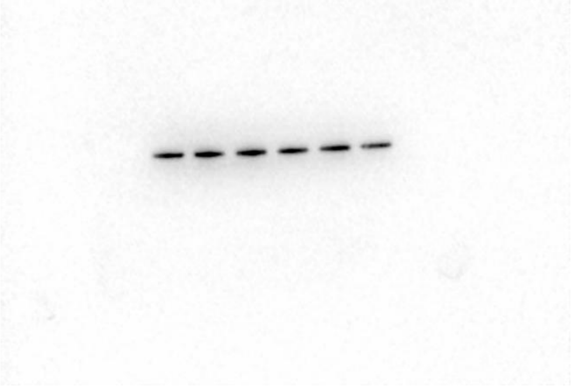

2C-NLRP3

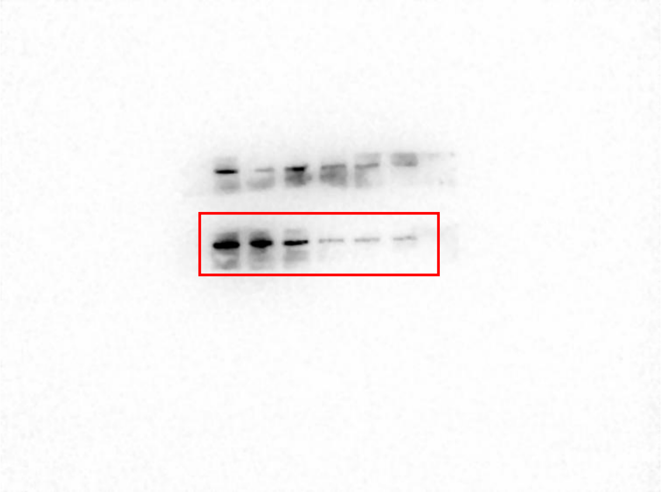

2C-ASC

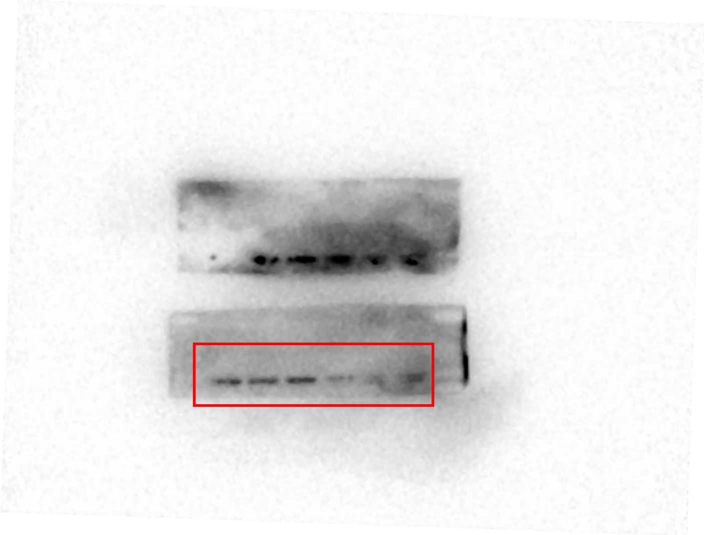

2C-Caspase-1

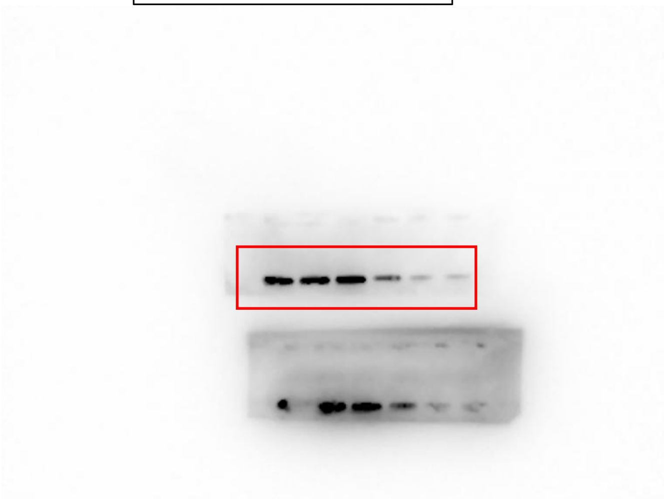

2C-IL-18

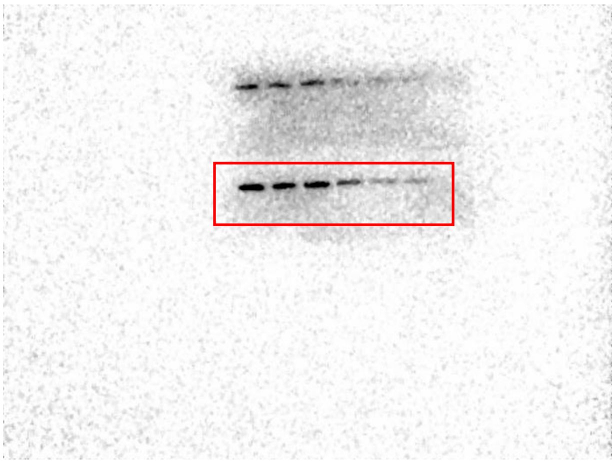

2C-IL-1 β

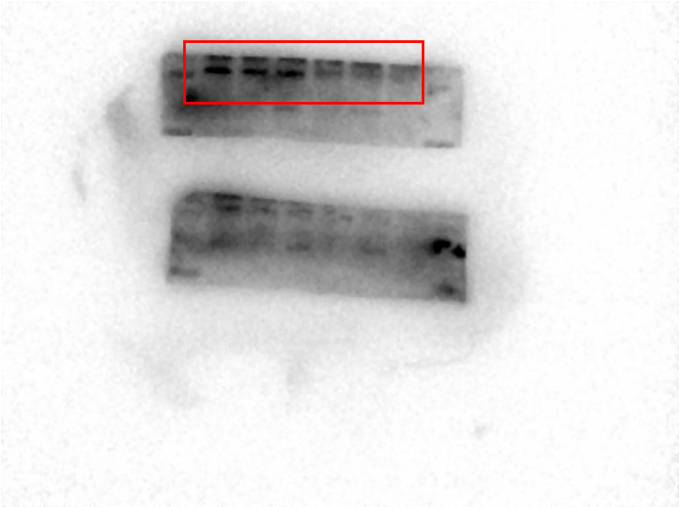

2C-GAPDH

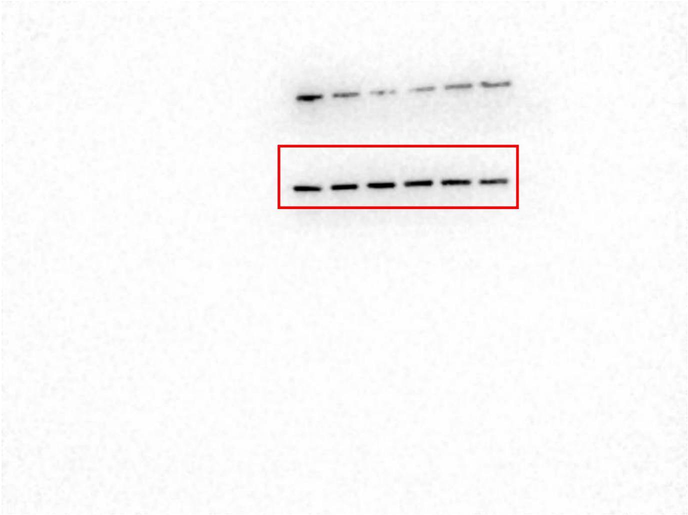

2D-NLRP3

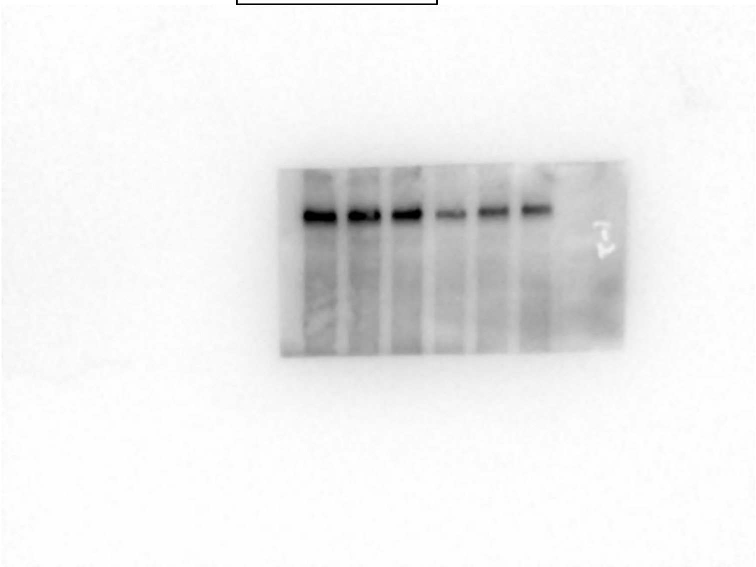

2D-ASC

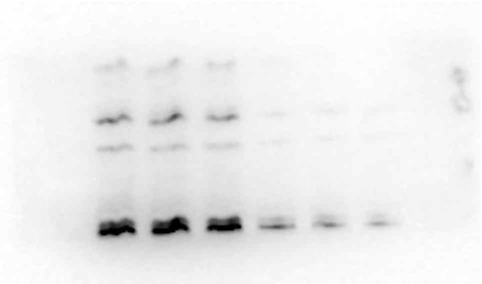

2D-Caspase-1

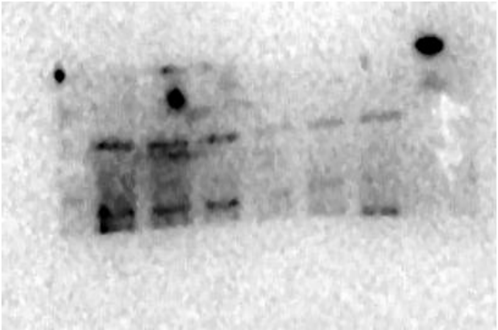

2D-IL-18

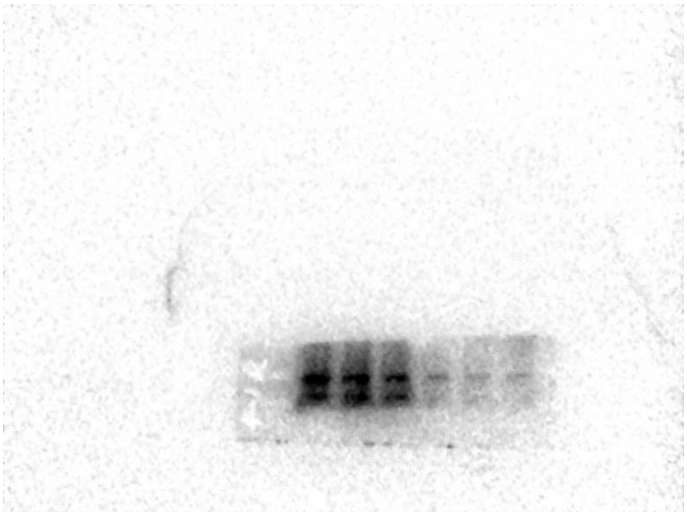

2D-IL-1 β

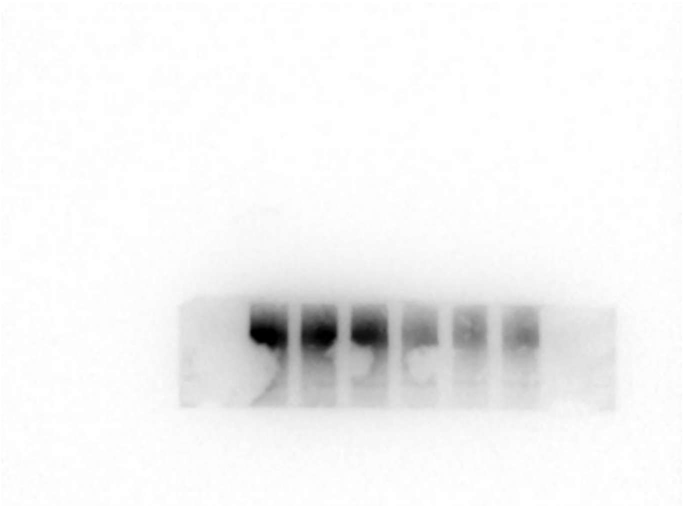

2C-GAPDH

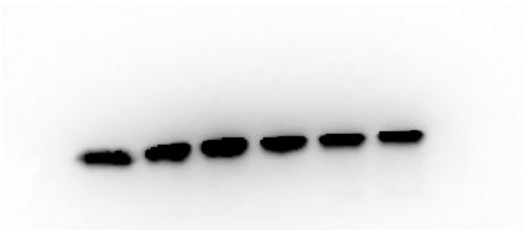

3B-NLRP3

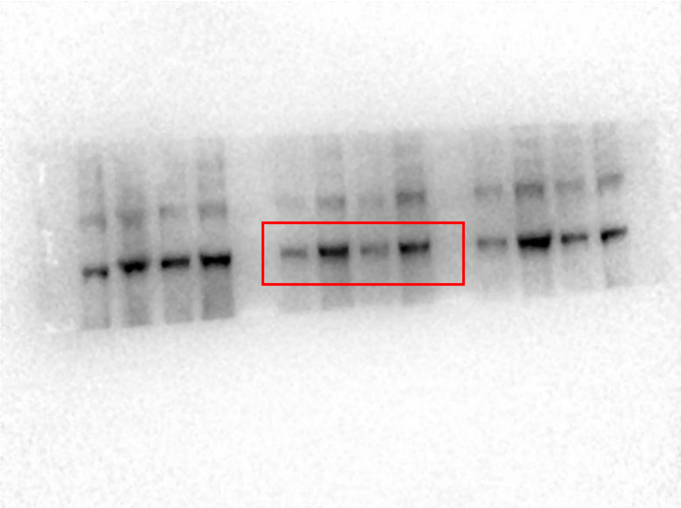

3B-ASC

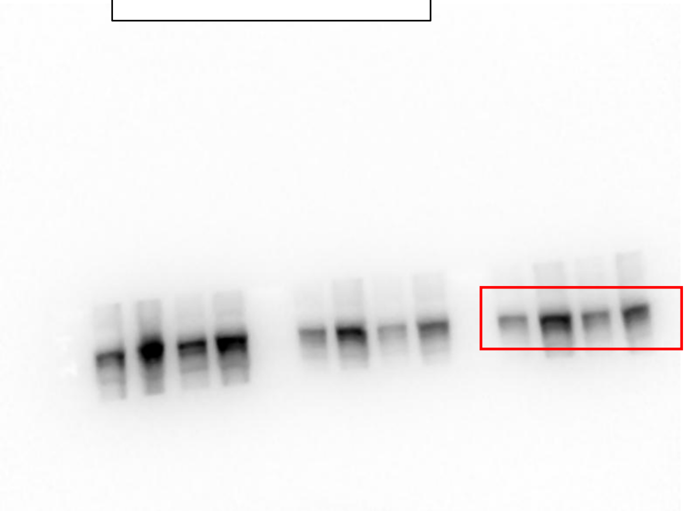

3B-Caspase-1

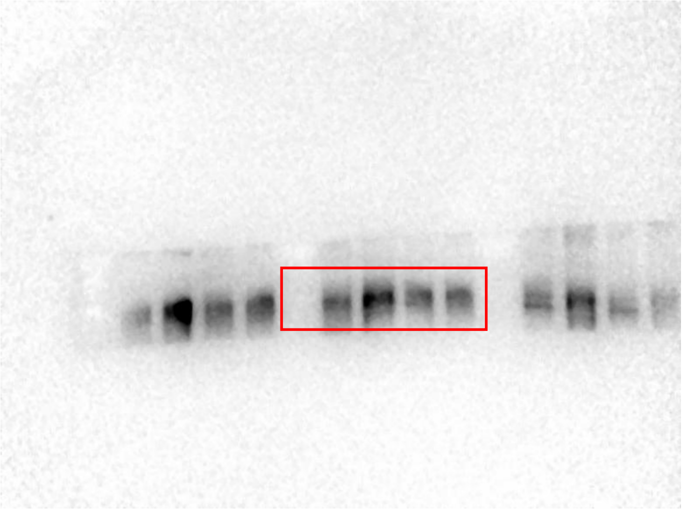

3B-IL-18

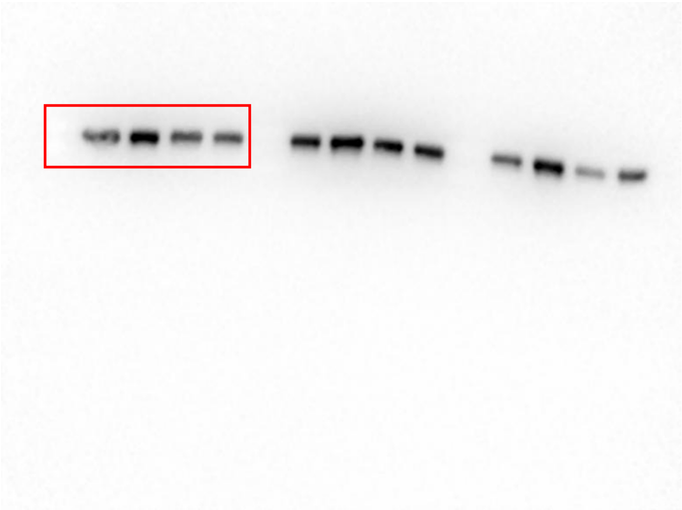

3B-IL-1 β

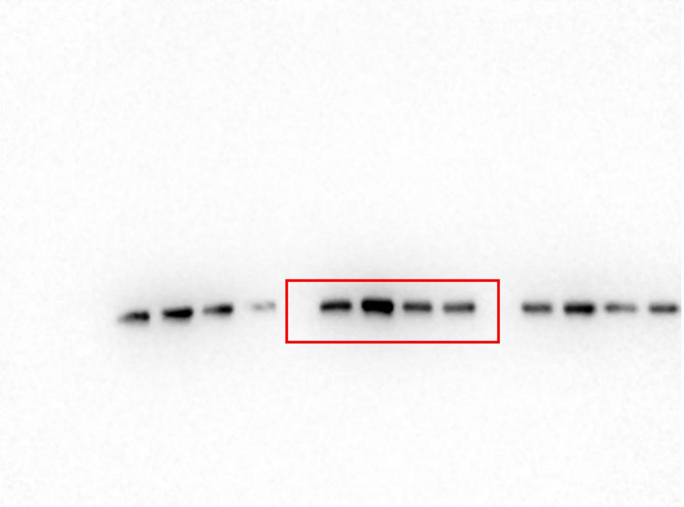

3B-GAPDH

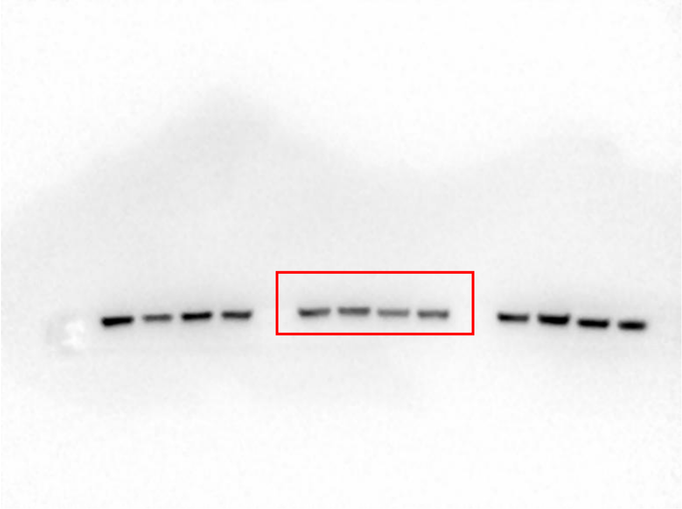

3C-Caspase-1

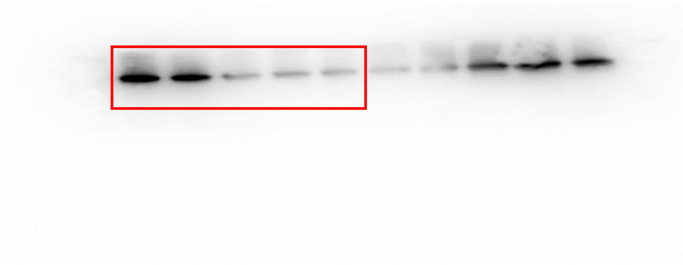

3C-GAPDH

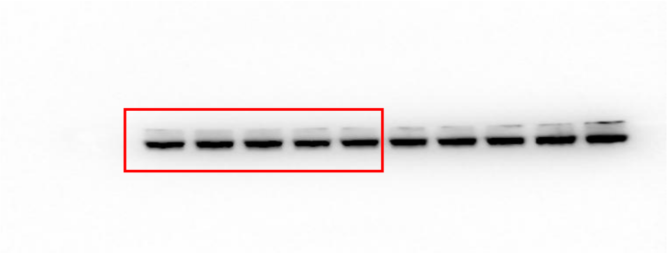

3D-NLRP3

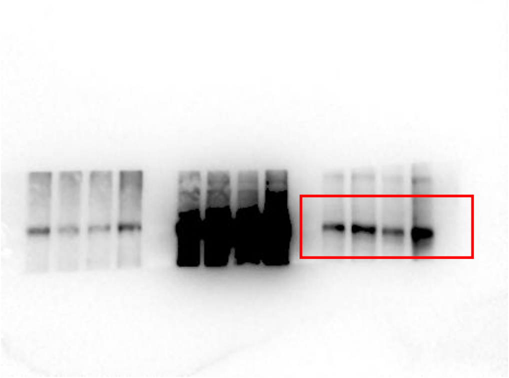

3D-ASC

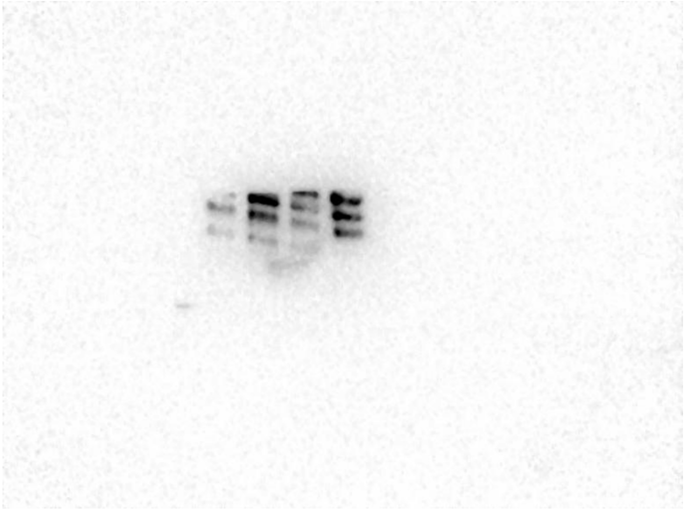

3D-Caspase-1

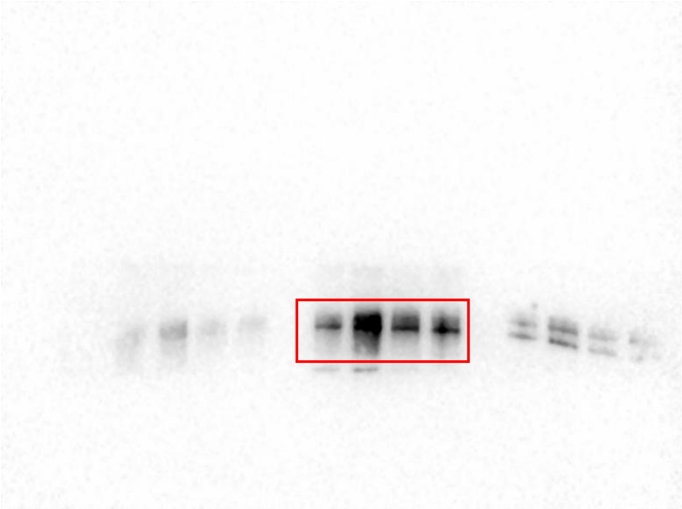

3D-IL-18

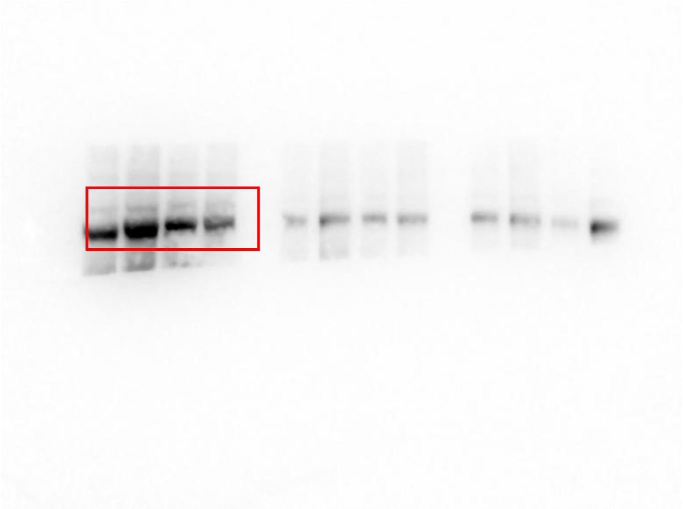

3D-IL-1 β

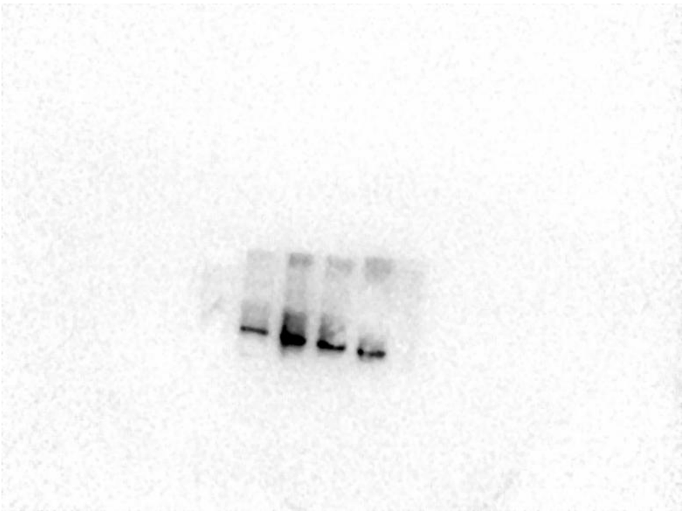

3D-GAPDH

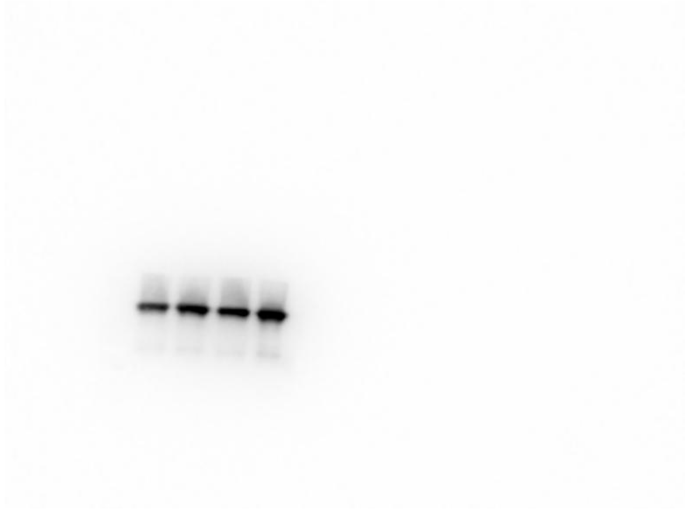

5B-FOXO3 α

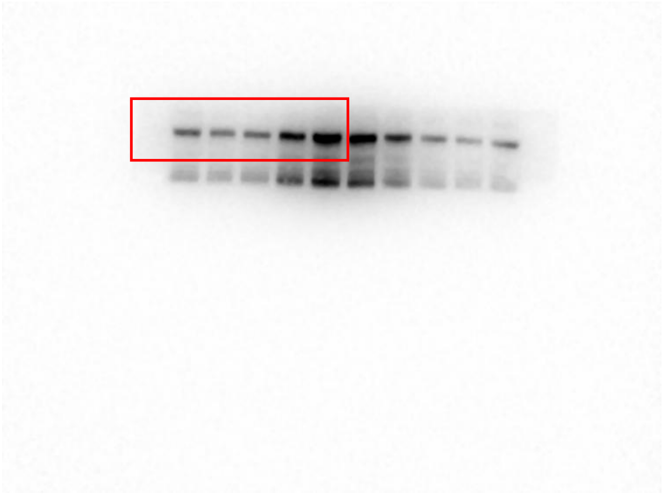

5B-GAPDH

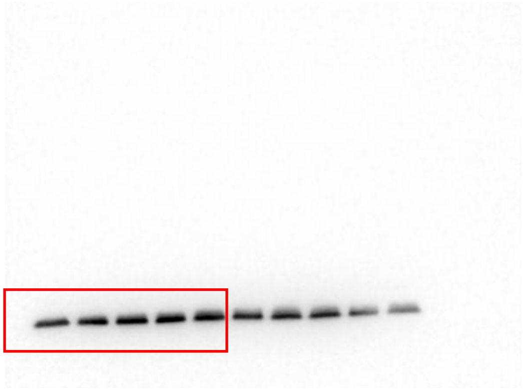

5F-4-HNE

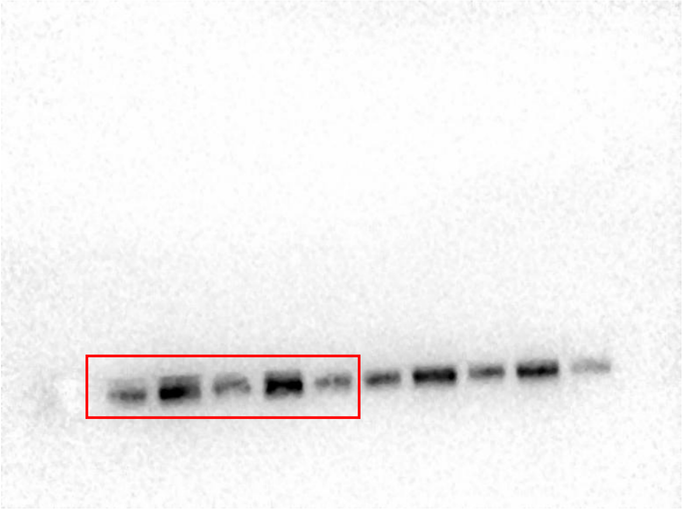

5F-Catalase

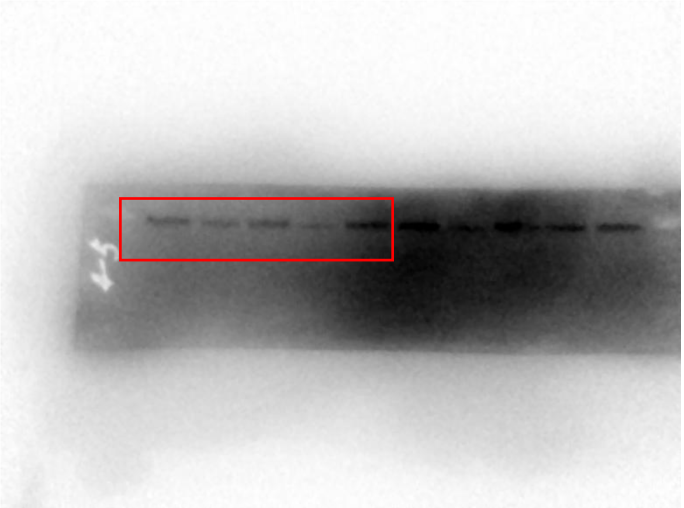

5F-GAPDH

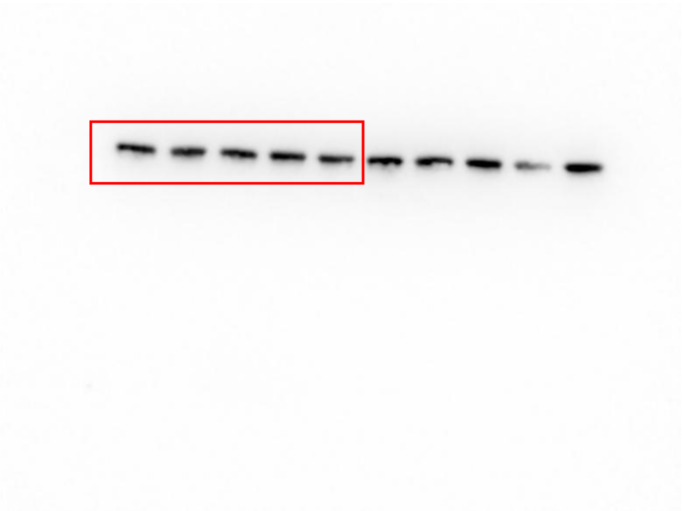

5G-FOXO3 α

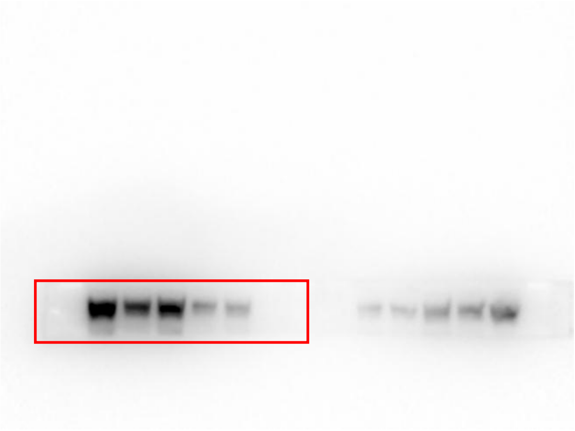

5G-SIRT3

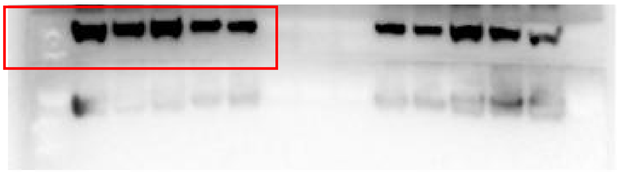

5G-mnSOD

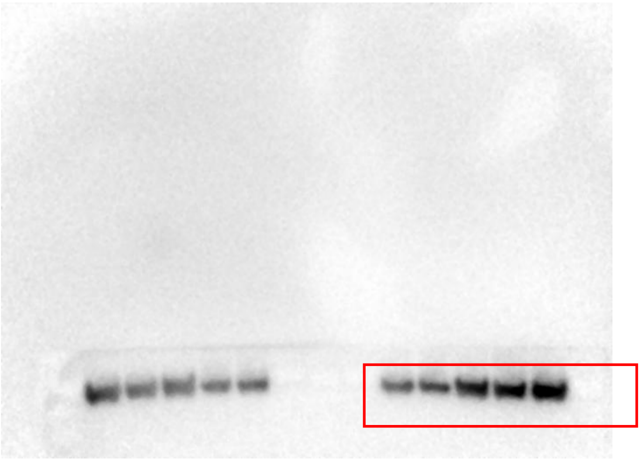

5G-GAPDH

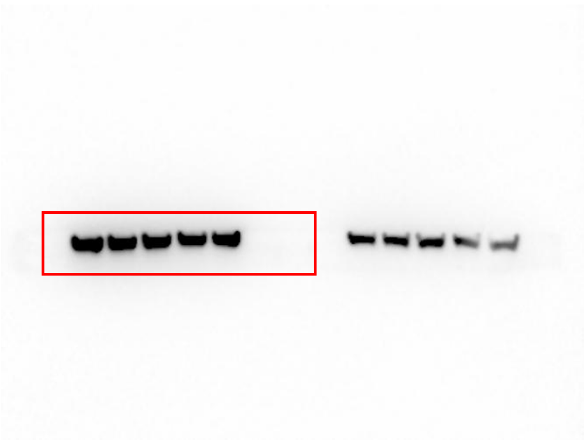

6A-GSDME

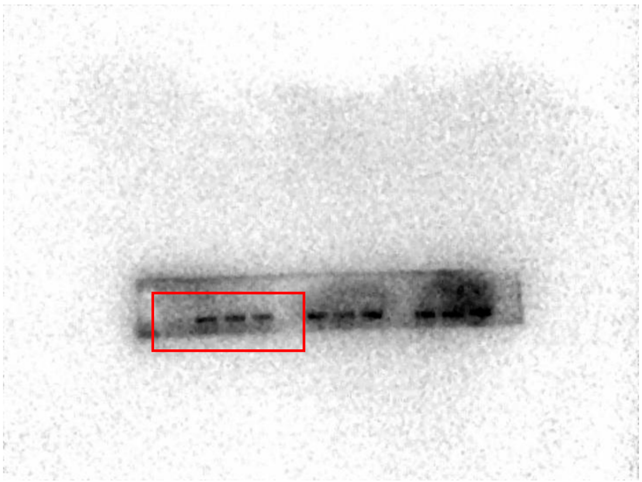

6A-GSDMD

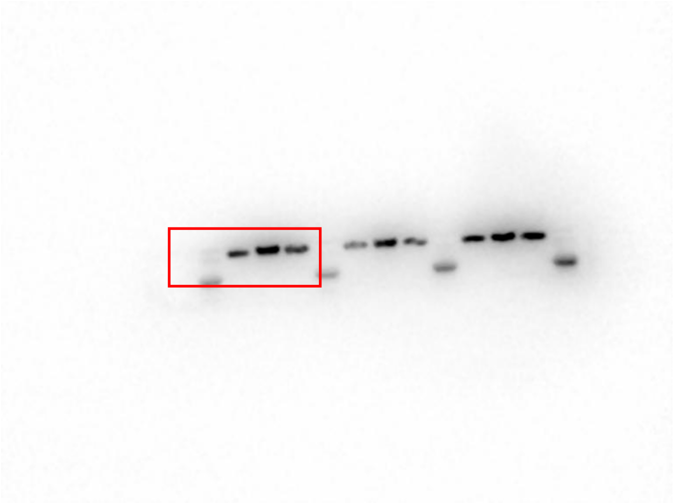

6A-GAPDH

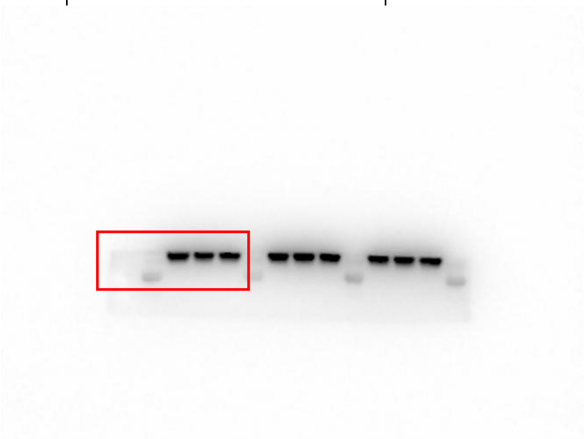

6C-Caspase-3

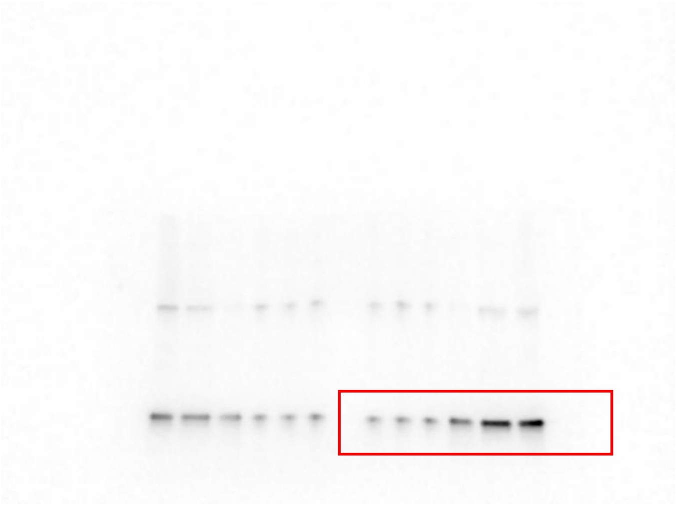

6C-Caspase-1

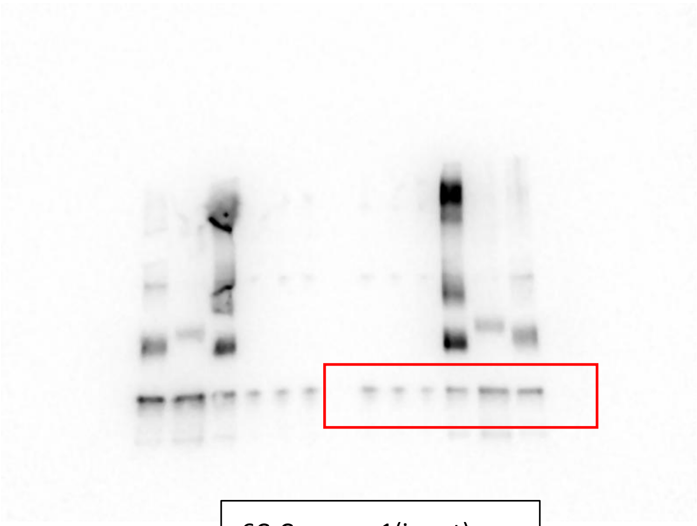

6C-Caspase-3(input)

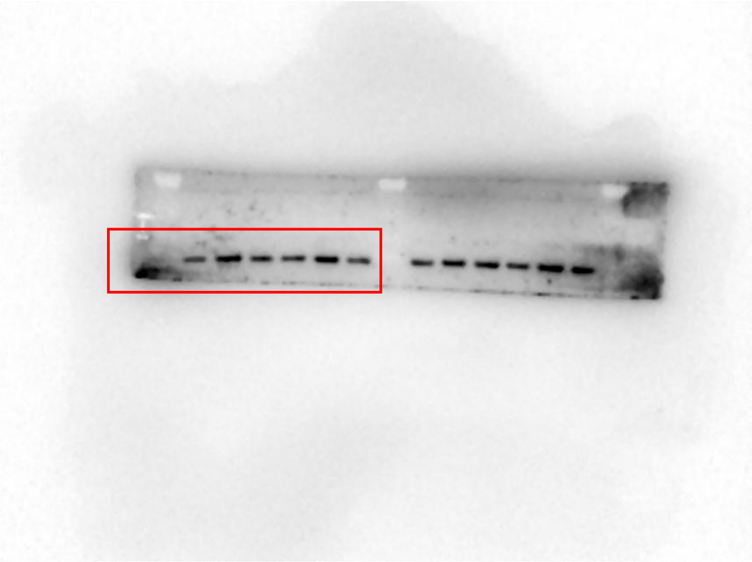

6C-Caspase-1(input)

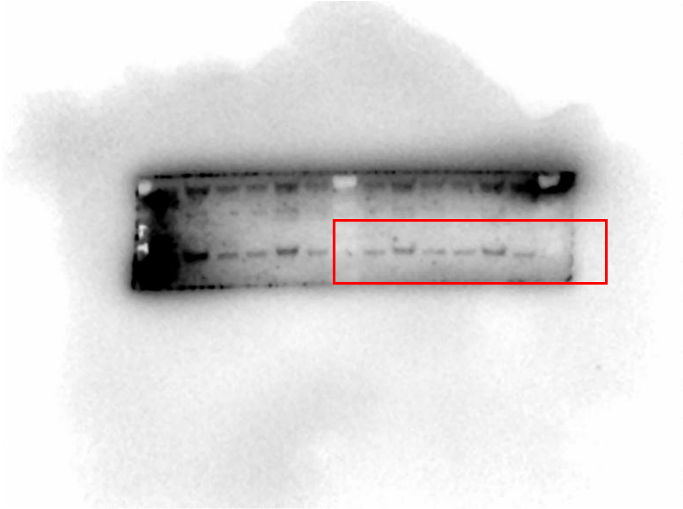

6C-GAPDH(input)

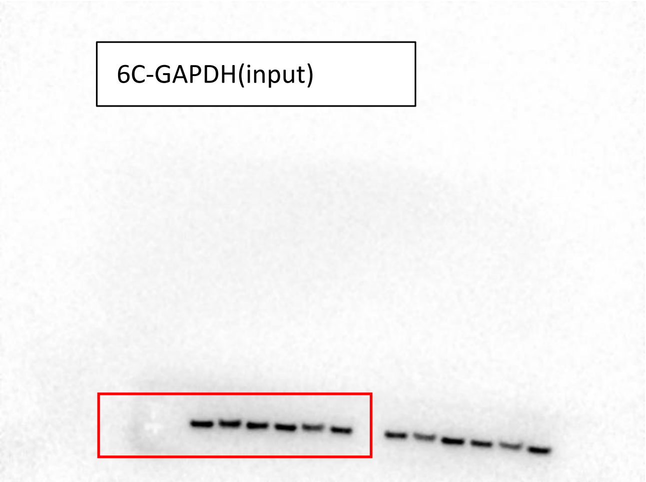

Supplement: Supplementary file 4 — Supplementary Material 4 [file 40659_2023_479_MOESM4_ESM.pdf]
